# Three additional new genera of acidocerine water scavenger beetles from the Guiana and Brazilian Shield regions of South America (Coleoptera, Hydrophilidae, Acidocerinae)

**DOI:** 10.3897/zookeys.855.33013

**Published:** 2019-06-13

**Authors:** Jennifer C. Girón, Andrew Edward Z. Short

**Affiliations:** 1 Department of Ecology & Evolutionary Biology, and Division of Entomology, Biodiversity Institute, University of Kansas, Lawrence, KS 66045, USA University of Kansas Lawrence United States of America

**Keywords:** aquatic beetles, new species, Neotropical region, taxonomy, seepage habitat

## Abstract

Recent study of the water scavenger beetle subfamily Acidocerinae in the Neotropical region has uncovered numerous undescribed species that are not able to be placed in existing genera. Here, we describe three new genera to accommodate 17 of these new species from South America: *Aulonochares***gen. nov.** for *Aulonochareslingulatus***sp. nov.** (French Guiana, Suriname), *Aulonocharesnovoairensis***sp. nov.** (Brazil), and *Aulonocharestubulus***sp. nov.** (Brazil, Guyana, Suriname, Venezuela); *Ephydrolithus***gen. nov.** for *Ephydrolithushamadae***sp. nov.** (Brazil), *Ephydrolithusminor***sp. nov.** (Brazil), *Ephydrolithusogmos***sp. nov.** (Brazil), *Ephydrolithusspiculatus***sp. nov.** (Brazil), and *Ephydrolithusteli***sp. nov.** (Brazil); and *Primocerus***gen. nov.** for *Primoceruscuspidis***sp. nov.** (Venezuela), *Primocerusgigas***sp. nov.** (Venezuela), *Primocerusneutrum***sp. nov.** (Guyana, Suriname, Venezuela), *Primocerusocellatus***sp. nov.** (Venezuela), *Primoceruspetilus***sp. nov.** (Brazil), *Primoceruspijiguaense***sp. nov.** (Venezuela), *Primocerusmaipure***sp. nov.** (Venezuela), *Primocerussemipubescens***sp. nov.** (Guyana), and *Primocerusstriatolatus***sp. nov.** (Suriname). The genus *Ephydrolithus***gen. nov.** is currently known to be restricted to seepages in the mountainous regions of the Brazilian Shield. *Aulonochares***gen. nov.** and *Primocerus***gen. nov.** are both currently only known from the Guiana Shield, though widespread in that region where they are associated with streams and seeps. We present differential diagnoses, maps, habitat details, and illustrations of all new genera and species here described.

## Abstract (es)

El estudio reciente de escarabajos acuáticos de la subfamilia Acidocerinae en la región neotropical, ha revelado numerosas especies no descritas que no pueden ser atribuidas a los géneros existentes. Aquí describimos tres géneros nuevos para acomodar 17 de estas especies nuevas de Suramérica: *Aulonochares***gen. nov.** para *Aulonochareslingulatus***sp. nov.** (Guyana Francesa, Surinam), *Aulonocharesnovoairensis***sp. nov.** (Brasil), y *Aulonocharestubulus***sp. nov.** (Brasil, Guyana, Surinam, Venezuela); *Ephydrolithus***gen. nov.** para *Ephydrolithushamadae***sp. nov.** (Brasil), *Ephydrolithusminor***sp. nov.** (Brasil), *Ephydrolithusogmos***sp. nov.** (Brasil), *Ephydrolithusspiculatus***sp. nov.** (Brasil), y *Ephydrolithusteli***sp. nov.** (Brasil); y *Primocerus***gen. nov.** para *Primoceruscuspidis***sp. nov.** (Venezuela), *Primocerusgigas***sp. nov.** (Venezuela), *Primocerusneutrum***sp. nov.** (Guyana, Surinam, Venezuela), *Primocerusocellatus***sp. nov.** (Venezuela), *Primoceruspetilus***sp. nov.** (Brazil), *Primoceruspijiguaense***sp. nov.** (Venezuela), *Primocerusmaipure***sp. nov.** (Venezuela), *Primocerussemipubescens***sp. nov.** (Guyana), y *Primocerusstriatolatus***sp. nov.** (Surinam). El género *Ephydrolithus***gen. nov.** se restringe hasta ahora a hábitats higropétricos en las regiones montañosas del Escudo Brasileño. *Aulonochares***gen. nov.** y *Primocerus***gen. nov.** son ambos hasta ahora conocidos del Escudo Guyanés, no obstante, ampliamente distribuidos en esa región en la que están asociados con corrientes y hábitats higropétricos. Presentamos diagnosis diferenciales, mapas, detalles sobre los hábitats e ilustraciones para todos los géneros y especies que aquí se describen.

## Introduction

The cosmopolitan subfamily Acidocerinae currently includes 16 genera, with eleven of these occurring in the Neotropical region (Short and Fikáček 2013, Minoshima et al. 2015, Girón and Short 2018). Until this century, the number of acidocerine lineages known from South America was relatively modest and their documented distribution quite spotty, particularly in the tropical areas of the continent. Recent fieldwork combined with renewed taxonomic efforts over the last two decades have revealed an eye-opening diversity of lineages and forms, resulting in the description of seven of the eleven presently recorded genera since 1999. And still, the discoveries continue unabated: an ongoing review of the Neotropical acidocerine fauna has revealed three additional new genera, which appear biogeographically restricted to the Brazilian and Guiana Shield regions of South America. Most of these new taxa occur in seepage habitats, which likely explains why they have remained hidden until now. Here we describe these three new genera to contain seventeen previously undescribed species.

## Materials and methods

Depositories of examined material:

**CBDG** Center for Biological Diversity, University of Guyana, Georgetown

**INPA** Instituto Nacional de Pesquisas da Amazônia, Manaus, Brazil (N Hamada)

**MALUZ** Museo de Artrópodos de la Universidad del Zulia, Maracaibo, Venezuela (J Camacho, M García)

**MIZA** Museo del Instituto de Zoología Agrícola, Maracay, Venezuela (L Joly)

**NZCS** National Zoological Collection of Suriname, Paramaribo (P Ouboter, V Kadosoe)

**SEMC** Snow Entomological Collection, University of Kansas, Lawrence, KS (A Short)

**USNM** US National Museum of Natural History, Smithsonian Institution, Washington, DC (C Micheli).

### Morphological methods

Nearly 280 specimens were examined. Specimen preparation and examination methods are identical to those given in Girón and Short (2017).

Descriptive sequence and morphological terminology largely follows Hansen (1991) except for the use of meso- and metaventrite instead of meso- and metasternum, and abdominal ventrites instead of abdominal sternites (see Lawrence and Ślipiński 2013). Terms for the ventral surface of the head follow Komarek (2004). Terminology for the metafurca follows Velázquez de Castro (1998; see also fig. 5C in Girón and Short 2017).

Descriptions of genera and species are organized in alphabetical order, whereas in the habitus figures species are grouped by similarity for ease of comparison. Maps were created using SimpleMappr (Shorthouse 2010). All specimen data which can be searched by species and/or collecting event are available online through the Collection Resources for Aquatic Coleoptera (CReAC) portal at http://creac.kubiodiversityinstitute.org/collections/.

## Results

**List of species and their known distribution**

*Aulonochares* gen. nov.

1. *Aulonochareslingulatus* sp. nov. French Guiana, Suriname

2. *Aulonocharesnovoairensis* sp. nov. Brazil (Amazonas)

3. *Aulonocharestubulus* sp. nov. Brazil (Roraima), Guyana, Suriname, Venezuela (Amazonas)

*Ephydrolithus* gen. nov.

4. *Ephydrolithushamadae* sp. nov. Brazil (Minas Gerais)

5. *Ephydrolithusminor* sp. nov. Brazil (Bahía)

6. *Ephydrolithusogmos* sp. nov. Brazil (Bahía)

7. *Ephydrolithusspiculatus* sp. nov. Brazil (Minas Gerais)

8. *Ephydrolithusteli* sp. nov. Brazil (Bahía, Minas Gerais)

*Primocerus* gen. nov.

9. *Primoceruscuspidis* sp. nov. Venezuela (Amazonas)

10. *Primocerusgigas* sp. nov. Venezuela (Amazonas)

11. *Primocerusneutrum* sp. nov. Guyana, Suriname, Venezuela (Bolívar)

12. *Primocerusocellatus* sp. nov. Venezuela (Amazonas)

13. *Primoceruspetilus* sp. nov. Brazil (Pará)

14. *Primoceruspijiguaense* sp. nov. Venezuela (Bolívar)

15. *Primocerusmaipure* sp. nov. Venezuela (Amazonas)

16. *Primocerussemipubescens* sp. nov. Guyana

17. *Primocerusstriatolatus* sp. nov. Suriname

### Taxonomy

#### Aulonochares gen. nov.

Taxon classificationAnimaliaColeopteraHydrophilidae

http://zoobank.org/B6E8B78C-3B5B-492E-A202-13E508E1799E

Figs 1 , 2 , 3 , 4

##### Type species.

*Aulonocharestubulus* sp. nov.

##### Differential diagnosis.

**Figure 1.**
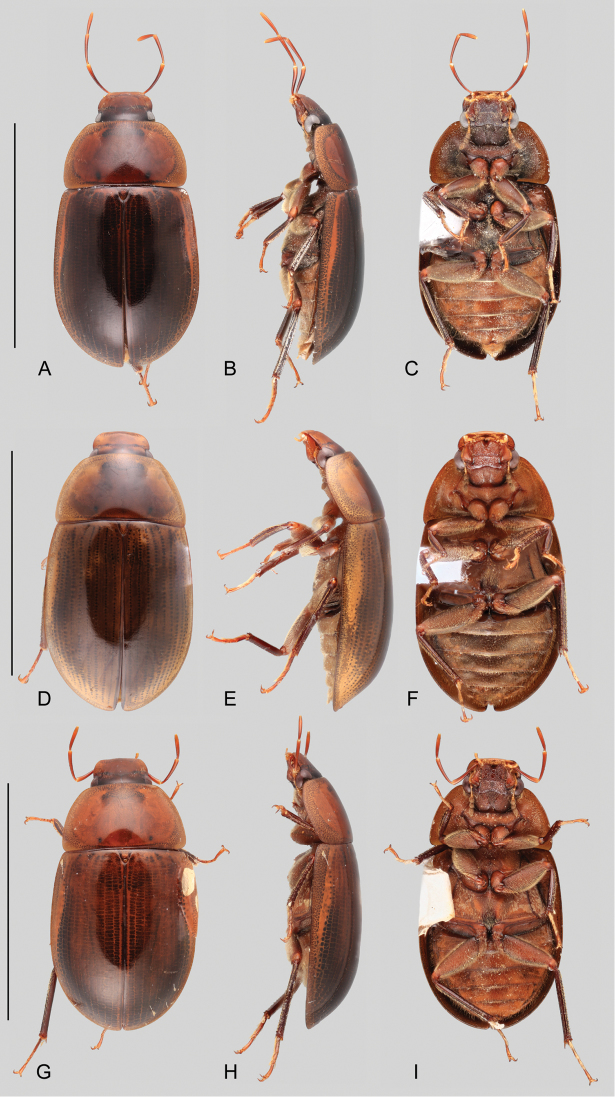
Habitus of *Aulonochares* spp.: **A–C***Aulonocharestubulus*: **A** dorsal view **B** lateral view **C** ventral view. **D–F***Aulonocharesnovoairensis*: **D** dorsal view **E** lateral view **F** ventral view. **G–I***Aulonochareslingulatus*: **G** dorsal view **H** lateral view **I** ventral view. Scale bars: 5 mm.

**Figure 2.**
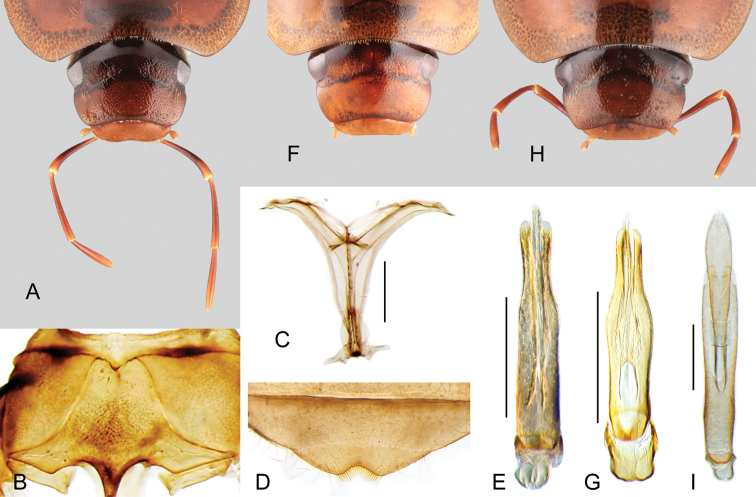
Head and internal structures of *Aulonochares* spp.: **A–E***Aulonocharestubulus*: **A** head, dorsal view **B** ventral view of mesoventrite with simply convex posterior elevation **C** posterior view of metafurca **D** fifth abdominal ventrite **E** aedeagus. **F, G***Aulonocharesnovoairensis*: **F** head, dorsal view **G** aedeagus. **H, I***Aulonochareslingulatus*: **H** head, dorsal view **I** aedeagus. Scale bars: 0.5 mm.

Medium sized beetles (5.8–7.5 mm), elongate oval in dorsal view, weakly convex in lateral view (see Fig. 1B, E, H). Color orange brown to dark brown; ventral surface covered with rather long golden setae, especially on abdominal ventrites. Head subquadrate in dorsal view (see Fig. 2A, F, H). Eyes relatively small. Clypeus with anterior margin only slightly narrower than posterior margin. Labrum fully exposed. Mentum and submentum roughly punctate (e.g., Fig. 1F). Antennae with nine antennomeres (e.g., Fig. 1C). Maxillary palps nearly 1.5 × longer than maximum width of head (e.g., Fig. 1A). Elytra without sutural striae, with net-like patterning visible throughout the entire surface (e.g., Fig. 1G); ground punctures and systematic punctures similar in size, shallowly impressed; serial punctures absent. Posterior elevation of mesoventrite simple, without carinae or ridges (Fig. 2B). Posterior femora glabrous at most along apical seventh. Ventral face of tarsomeres 1–4 densely covered by stiff setae. Apex of fifth abdominal ventrite strongly emarginate; emargination fringed by stout setae (Fig. 2D). Aedeagus (Fig. 2E, G, I) somewhat cylindrical, with parameres forming a 5–7 × longer than wide tube; basal piece very short and strongly concave.

*Aulonochares* can be easily mistaken for *Helochares*, especially in the field, based on overall body size, shape and coloration, number of antennomeres and apical emargination of the fifth ventrite. *Aulonochares* can be distinguished from other Neotropical acidocerines by the following unique combination of characters: head subquadrate in shape (clypeus with anterior margin only slightly narrower than posterior margin; as opposed to head rather trapezoidal, with anterior margin of clypeus conspicuously narrower than its posterior margin as in Neotropical *Helochares*); eyes relatively small, separated by a distance nearly 6.5 × the maximum width of an eye (as opposed to eyes of moderate size, separated by approximately 4 × the width of one eye as in *Helochares* (see Hansen 1991: 150)); mentum and submentum roughly punctate (submentum usually rather smooth in Neotropical *Helochares*); pubescence covering abdominal ventrites composed of long golden setae (short setae in Neotropical *Helochares*); ventral surface of tarsomeres 1–4 densely setose (tarsomeres bearing two longitudinal rows of denticles in Neotropical *Helochares*); aedeagus narrow and tubular in shape.

##### Description.

Medium sized beetles, total body length 5.8–7.5 mm, width 3.1–4.0 mm; body elongate oval, weakly convex in lateral view (see Fig. 1B, E, H), orange brown to dark brown in color (Fig. 1), slightly paler on labrum, labial palpi, along lateral margins of pronotum and elytra, on ventral surface (including abdominal ventrites), and tarsi; body setae, including hydrofuge pubescence, setae of systematic punctures, and especially on abdominal ventrites, golden and rather long; hydrofuge pubescence on surface of femora denser, with shorter setae. ***Head.*** Subquadrate in dorsal view, with lateral margins seemingly constricted at anterior margin of eyes (Fig. 2A, F, H). Frons and clypeus with moderately marked ground punctures, irregularly and rather densely distributed over the surface, accompanied by scattered seta-bearing systematic punctures, longer and denser on antero-lateral areas of frons and along anterior area of clypeus; surface between punctures smooth and shiny. Frons transversely impressed by anterior margin of pronotum. Frontoclypeal and midcranial sutures well defined, visible as complete, fine grooves; distance between inner anterior corner of eye and frontoclypeal suture approximately 0.5 × maximum length of eye. Clypeus with lateral margins slightly convex, anterior corners roundly angulate, forming a nearly straight angle; anterior margin of clypeus widely roundly emarginate, only slightly narrower than posterior margin. Eyes relatively small and subquadrate in dorsal view; maximum length of eye 0.5 × distance between anterior margin of eye and anterior margin of clypeus; distance between eyes nearly 6.5 × maximum width of eye. Labrum wide, fully exposed, nearly half as long, and collinear to perpendicular to clypeus; dorsal surface only slightly convex, with scattered fine punctures and few systematic punctures; anterior margin only slightly sinuate, mesally slightly roundly bent inwards, with few denticles along emargination; anterior corners with few setae. Temporae slightly concave, densely covered by rather long and relatively thick setae (hydrofuge pubescence); posteroventral area rather strongly produced. Gular sutures opposite, semicircular, with surface slightly elevated and shiny. Surface of gula and postgenae covered by long fine setae. Mentum (e.g., Fig. 1F) parallel sided, with lateral margins fringed by golden setae; surface coarsely punctate, with punctures somewhat obliquely directed; anterior margin with deep U-shaped emargination, sometimes marked by a carina; surface distad of emargination perpendicular to ventral surface of head, smooth, concave, and dorsally directed. Submentum as elevated plate, coarsely punctate, with scattered setae; posterior margin as a low, sinuate, wide ridge; well-developed ocular ridge (e.g., Fig. 1F). Maxilla with ventral surface of cardo and stipes with scattered punctures and setae; outer dorsal margin of palpifer with few stiff, spiniform setae; limit between cardo and stipes oblique; maxillary palps curved inward, orange brown, longer than antennae, nearly 1.5 × longer than maximum width of head (e.g., Fig. 1A); each palpomere paler towards its apex; apex of palpomere 3 bearing sensilla. Mandibles with apex bifid (examined in *A.tubulus*). Labial palps yellowish, nearly as long as maximum length of mentum, dorsoventrally flattened; palpomere 2 with outer margin only slightly convex near apex, with several long setae around midlength and at apex; palpomere 3 obovate, with a long subapical seta on outer corner. Antennae (e.g., Fig. 1C) with nine antennomeres, paler (yellowish) than general coloration of head; antennomere 1 reaching anterior third of ventral surface of eye (reaching midlength of cardo), nearly 2.5 × longer than antennomere 2, with outer surface densely covered by setae; antennomere 2 thicker, and nearly as long as antennomere 3; antennomere 3 cylindrical, 4 and 5 trapezoid; antennomere 6 forming a well differentiated, asymmetric cupule; antennomeres 7–9 slightly flattened, forming a loosely articulated, pubescent club, with antennomeres 7 and 8 similar in shape and length, and antennomere 9 1.5 × longer than 7; apex of antennomere 9 with a few longer setae compared to general pubescence of club. ***Thorax.*** Pronotum widest at base, narrowed anteriorly, surface evenly convex, with internal structural reticulations visible along lateral areas; ground punctation shallow, uniformly sparse, with surface between punctures smooth and shiny; seta-bearing systematic punctures forming paired anterolateral semicircles; anterior margin of pronotum fringed by short, rather sparse setae; lateral and anterior areas of pronotum translucent, with inner reticulations. Scutellar shield of moderate size, triangular, posteriorly rounded, nearly as long as wide, with punctation as in pronotum. Prosternum (e.g., Fig. 1I) nearly as long as half the length of a procoxa; anterior margin of prosternum mesally projected as a wide triangle, slightly carinate along longitudinal midline; surface of median area of prosternum slightly elevated, somewhat densely covered by rather long, fine setae; intercoxal process projected from posterior margin of procoxal cavities, rectangularly shaped in outline, mesally longitudinally carinate. Mesoventrite (Fig. 2B) not fused to mesepisterna, densely setose for the most part, with posterolateral smooth and glabrous areas; anterior margin nearly 0.3 × as wide as anterior margin of mesepisternum; anterior rib of mesoventrite bearing paired oblique to parallel pearlescent maculae; posterior elevation of mesoventrite simply convex, without carinae or ridges (Fig. 2B); mesepisternum with surface nearly flat, densely covered by fine setae; mesepimeron trapezoid, with densely pubescent surface. Mesofurca (examined in *A.tubulus*) with short arms, 0.7 × length of mesocoxae; apical half of arms free, somewhat triangular at apex. Metaventrite mesally elevated, narrowly anteriorly, widely and flat posteriorly; surface of metaventrite densely and uniformly pubescent; mesal region of posterior margin rounded to truncate. Metepisterna approximately 3 × longer than wide, with posterior margin oblique. Metepimeron triangular, elongate to short. Metafurca (examined in *A.tubulus*, Fig. 2C) 1.46 × wider than long, with furcal arms as long as stalk; stalk triangular (wider near the crux, gradually narrowing ventrally), with paired longitudinal keels extending along basal third of posterior face, fusing together towards crux; with a well-developed median keel on anterior face extending to anterior margin of dorsal sheets; outer margins of stalk diverging towards crux, more strongly so along basal third; each furcal arm sickle-shaped, with apex (hemiductus) explanate in dorsal view, with apical region sinuate, pointing laterally; anterior tendons inserted nearly at mid length of dorsal edge of furcal arms; well-developed dorsal sheaths, wider than widest point of lateral sheaths. ***Elytra.*** Surface even (without elevations or depressions) and smooth, without sutural striae; ground punctures and systematic punctures very shallowly marked, all similar in size and degree of impression, and evenly distributed across surface; seta-bearing systematic punctures rather scarce, at most only distinguishable as rows along midline, third outer fourth, and near outer margin of each elytron, more evident along posterior fourth; serial punctures absent; elytral margins slightly flared; net-like patterning visible throughout the entire surface, especially along outer margins (e.g., Fig. 1G), with a pale lateral band extending from anterior margin up to apical third on each elytron. Epipleura well-developed, surface flat, with sparse fine setae and irregular sculpture, anteriorly wide, gradually narrowing posteriorly, extending up to line of posterior margin of first abdominal ventrite; inner margin of epipleura at most slightly bent at anterior outer corner of metepisternum; well-developed pseudepipleura, perpendicularly positioned, nearly as wide as anterior portion of epipleura, extending along entire outer margin of elytra, with rather smooth surface. Hind wings well developed. ***Legs.*** All coxae, trochanters and femora with dense pubescence, except on (at most) apical seventh of femora, in which surface is mostly glabrous, shiny and slightly reticulated. Anterior surface of mesocoxae with interspersed small denticles. All femora antero-posteriorly flattened; metafemora with rather well-developed tibial grooves, at most glabrous along apical seventh. Tibiae slender, cylindrical; spines forming longitudinal rows along tibiae rather small, accompanied by conspicuous and somewhat dense golden setae; protibiae with median longitudinal row of small, appressed spines along anterior surface; apical spurs of protibiae very short (not exceeding length of tarsomere 1) and stout; apical spurs of metatibiae asymmetrical, inner posterior spur largest, nearly as long as metatersomere 1, 2 × longer than shorter spur (inner anterior). All tarsi with five tarsomeres, bearing numerous long hair-like setae on dorsal face, and densely covered by stiff setae on ventral face of tarsomeres 1–4; pro- and mesotarsomeres 1–4 similar in size and shape, with tarsomere 5 approximately as long as tarsomeres 2–4 combined, with few setae on ventral face; metatarsomeres 2, 3+4, and 5 similar in length; metatarsomere 4 shortest; claws rather large, curved; well-developed empodium, bearing a pair of long, curved apical setae. ***Abdomen.*** Abdomen with five ventrites, all uniformly and rather densely covered by fine and rather long, fine, golden setae, particularly longer along lateral margins; first ventrite medially convex, remainder ventrites rather flat; posterior margin of fifth ventrite with a medial triangular emargination, fringed by thick, flat spine-like setae (Fig. 2D); ninth tergite with transverse V-shaped impression, lateral margins deeply emarginate near midlength, and posterior margin rounded to mesally emarginate; ninth ventrite as fully sclerotized plate, with lateral margins posteriorly diverging, and posterior margin widely, roundly emarginate. Aedeagus (Fig. 2E, G, I) with well-developed basal piece, 0.1–0.25 × the length of parameres, longitudinally strongly convex; parameres basally fused together into a rather cylindrical tube, 5–7 × longer than wide, with basal margin rounded to truncate, and lateral margins straight to sinuate; median lobe nearly as long as parameres, with well-developed lateral basal apodemes; median lobe rounded at apex, either as a narrow tube throughout, or tongue-like and distally widened; gonopore reduced (inconspicuous), situated near apex of median lobe.

##### Larvae.

The immature stages are unknown.

##### Etymology.

Named from the Greek *aulon*, meaning pipe, tube, in reference to the unique tubular shape of the aedeagus of the species in the genus, combined with the ending -*chares*, as a reference to the general similarity with *Helochares* in the Acidocerinae. To be treated as masculine.

##### Distribution.

**Figure 3.**
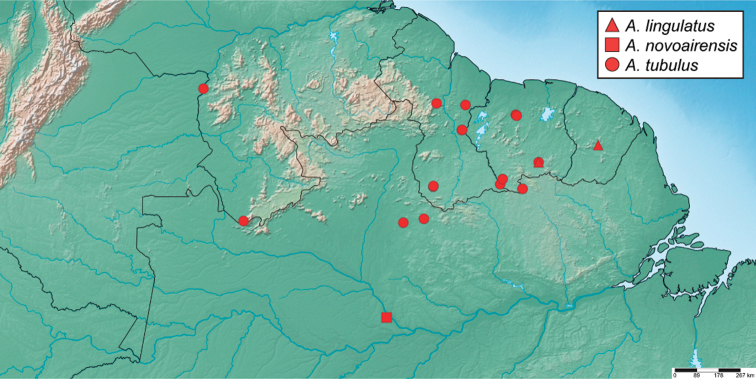
Distribution of *Aulonochares* spp.

To date known only from the Guiana Shield region of South America, where it is broadly distributed from southern Venezuela to French Guiana (Fig. 3).

##### Remarks.

**Figure 4.**
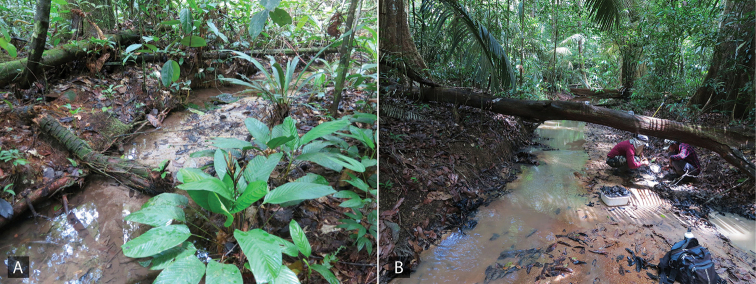
Habitat of *Aulonochares* spp. **A** habitat and type locality for *A.lingulatus*, Suriname: Kasikasima, collecting event SR12-0320-02A **B** habitat for *A.tubulus*, Guyana: Upper Berbice, collecting event GY14-0921-03H.

All known species are associated with small forested streams, typically with sand and detritus substrate where they are found along the margins (see Fig. 4). Adult females of *Aulonocharestubulus* have been observed to carry their egg case attached to the ventral side of their abdomen as other closely-related genera such as *Helochares* and *Helobata*.

### Characters of taxonomic importance for *Aulonochares*

The external morphology of *Aulonochares* is extremely uniform across species.

**Coloration.** Even though coloration is not typically a reliable diagnostic feature in acidocerines, the dorsal coloration in *Aulonochares* is helpful for recognizing the species described here: *A.tubulus* is typically dark brown, *A.lingulatus* is orange brown and *A.novoairensis* is yellowish brown (see Fig. 1). Because teneral specimens may appear paler, the color of a specimen should not alone be considered as diagnostic. Specimens that have been extracted for DNA become uniformly dark brown in coloration.

**Aedeagus.** In all the known species of *Aulonochares* the aedeagus has basally fused parameres forming a tube which is 5–7 × longer than wide and becomes dorsoventrally flattened along the apical half; the median lobe is either cylindrical or broad and flat, and can slide within the parameres, so its extension beyond the apex of the parameres cannot be considered a diagnostic feature to distinguish species. The apex of the parameres can also be cylindrical or flattened. The basal piece is very short. The general form of the aedeagus of *Aulonochares* is unique among the Acidocerinae.

### Key to the species of *Aulonochares*

**Table:** 

1	General coloration orange brown (Fig. 1G–I); median lobe of aedeagus broad and flat, wider than apical portion of a paramere (Fig. 2I)	*** A. lingulatus ***
–	General coloration dark brown to yellowish brown; median lobe of aedeagus cylindrical, as wide as apical portion of a paramere	**2**
2	General coloration dark brown (Fig. 1A–C); aedeagus parallel sided along basal 2/3 (Fig. 2E)	*** A. tubulus ***
–	General coloration yellowish brown (Fig. 1D–F); aedeagus widened at 2/3 (Fig. 2G)	*** A. novoairensis ***

#### Aulonochares lingulatus sp. nov.

Taxon classificationAnimaliaColeopteraHydrophilidae

http://zoobank.org/F03E9C40-595C-4EAA-B84A-37EAC2B753D3

Figs 1G–I , 2H, I , 3 , 4A

##### Type material.

**Holotype** (♂): “SURINAME: Sipaliwini District; 2.97731N, 55.38500W; 200 m; Camp 4 (low), Kasikasima; sandy stream on trail to METS camp; 20.iii.2012; leg. A. Short; SR12-0320-02A” (NZCS). **Paratypes (12): FRENCH GUIANA**: “Unnamed Trib. To Crique Nouvelle France, 3.59627N, 53.17637W, above Courant double; 09.xi.2016, leg. D. Post” (SEMC, 1, DNA voucher specimen SLE 1173). **SURINAME: Sipaliwini District**: Same data as holotype (NZCS, SEMC, 10, including DNA voucher SLE 415); same, except “sandy creek, trail to Kasikasima; flotation; 22.iii.2012; SR12-0322-02A” (SEMC, 1).

##### Differential diagnosis.

*Aulonochareslingulatus* can be distinguished by its orange brown general coloration (Fig. 1G–I), and the shape of the median lobe of aedeagus, which is broad and flat (Fig. 2I).

##### Description.

Body length 6.0–6.5 mm, width 3.2–3.6 mm. General coloration

orange brown (Fig. 1G–I). Aedeagus (Fig. 2I) with outer margins of parameres subparallel, slightly diverging apically; median lobe flat, gradually widening towards apical region, widely rounded at apex.

##### Etymology.

Named with the Latin word *lingulatus* meaning tongue-like, after the shape of the median lobe of the aedeagus in this species.

##### Distribution.

*Aulonochareslingulatus* is known from the area surrounding Mt. Kasikasima in Suriname and a locality in central French Guiana (Fig. 3).

##### Remarks.

All specimens were collected in densely forested sandy streams (Fig. 4A).

#### Aulonochares novoairensis sp. nov.

Taxon classificationAnimaliaColeopteraHydrophilidae

http://zoobank.org/F78999DD-14F1-405C-9262-DA883BDFB1F3

Figs 1D–F , 2F, G , 3

##### Type material.

**Holotype** (♂): “BRAZIL: Amazonas: Novo Airão; 2°41'2.2878"S, 60°56'18.24"W; 60 m; detrital pools in forest along sides of blackwater creek; 9.vi.2017; leg. Benetti; BR17-0609-04B” (INPA). **Paratype (1)**: Same data as holotype (SEMC, DNA voucher specimen SLE 1268).

##### Differential diagnosis.

*Aulonocharesnovoairensis* can be distinguished by its yellowish brown general coloration (Fig. 1D–F), and the shape of the aedeagus, which is widened at 2/3, with cylindrical median lobe (Fig. 2G).

##### Description.

Body length 6.3–6.9 mm, width 3.4–3.6 mm. General coloration yellowish brown (Fig. 1D–F). Aedeagus (Fig. 2G) with outer margins of parameres sinuate, widest along 2/3; median lobe cylindrical, somewhat acute at apex.

##### Etymology.

Named after Novo Airão municipality in the state of Amazonas in Brazil.

##### Distribution.

Currently only known from a single locality in the central Amazon near Manaus (Fig. 3).

##### Remarks.

The single collection of this species was from densely forested, shallow detrital pools immediately adjacent to a blackwater stream.

#### Aulonochares tubulus sp. nov.

Taxon classificationAnimaliaColeopteraHydrophilidae

http://zoobank.org/B79D0338-3EE9-4B12-8357-20006B091877

Figs 1A–C , 2A–E , 3 , 4B

##### Type material.

**Holotype** (♂): “SURINAME: Sipaliwini District; 2°00.342'N, 55°58.149'W; 337 m; Sipaliwini Savanna nature Res., 4-Brothers Mts.; clearwater stream, sandy with emergent vegetation; at night; 31.iii.2017; leg. A. Short; SR17-0331-01F” (NZCS). **Paratypes (156): BRAZIL: Roraima**: “00°46'35.1"N, 60°19'58.7"W; 97 m; Rorainópolis, Recanto da Cachoeira, vicinal 12; creek flowing through gallery forest; 10.1.2018; leg. A. Short; BR18-0110-04A” (SEMC, 3); “00°54.786'N, 59°34.397'W; 150 m; Caroebe, Rio Caroebe, ca. 13 Km NE of Caroebe; margins of sandy river; 17.i.2018; leg. A. Short & Benetti; BR18-0117-04A” (SEMC, 1). **GUYANA: Region 9**: “2°05.095'N, 59°14.174'W; 250 m; Parabara, trail to mines; detrital pools in forest; 2.xi.2013; leg. Short, Isaacs, Salisbury; GY13-1102-01A” (CBDG, SEMC, 8); same, except “2°06.492'N, 59°13.653'W;274 m; Parabara, N side of river; small flowing forested creek, detritus margins & leaf packs; 3.xi.2013; GY13-1103-02A” (SEMC, 2). **Region 8**: “5°07.539'N, 59°06.732'W; 80 m; Konawaruk River, basecamp 2 (NARIL basecamp); unnamed clearwater creek, slow flowing, shallow; 15.ix.2014; leg. Salisbury & La Cruz; GY14-0915-02” (SEMC, 6). **Region 6**: “4°09.143'N, 58°11.207'W; 105 m; Upper Berbice, c. 1 Km W Basecamp 1; small sandy stream; 21.ix.2014; leg. A. Short; GY14-0921-03A” (SEMC, 2); same, except “margins of creek; 22.iv.2014; leg. Short, Salisbury, La Cruz; GY14-0921-03H” (SEMC, 4); same, except “4°09.136'N, 58°11.365'W; 106 m; Upper Berbice, ca. 1.1 Km W of basecamp 1; stream detrital pool; 23.ix.2014; GY14-0923-02A” (SEMC, 1); same, except “4°09.289'N, 58°10.717'W; 95 m; Upper Berbice, Basecamp 1; margins of basecamp creek; 24.ix.2014; GY14-0924-01A” (SEMC, 1); same, except “4°09.241'N, 58°10.627'W; 109 m; puddles along road; GY14-0924-02A” (SEMC, 4); same, except “margins of creek with leaf packs and mud; 25.ix.2014; leg. Short & La Cruz; GY14-0925-01B” (SEMC, 1); same, except “detritus pools in dry creekbed; leg. Short, Salisbury, La Cruz; GY14-0925-01D” (SEMC, 1); same, except “5°03.892'N, 58°03.303'W; 71 m; Upper Berbice, Logging Road Km 1; marsh and creek; 29.ix.2014; GY14-0929-01B” (CBDG, SEMC, 12). **SURINAME: Sipaliwini District**: “2°10.521'N, 56°47.244'W; 228 m, Camp 1, on Kutari River; forest swamp; 22.viii.2010; leg. Short & Kadosoe; SR10-0822-02A; 2010 CI-RAP Survey” (SEMC, 2); same, except “2°21.776'N, 56°41.861'W; 237 m; Camp 3, Wehepai; sandy forest creek; 4–6.ix.2010; SR10-0904-01A” (SEMC, 7); “2.97731N, 55.38500W; 200 m; Camp 4 (low), Kasikasima; sandy stream on trail to METS camp; 20.iii.2012; leg. A. Short; SR12-0320-02A; 2010 CI-RAP Survey” (SEMC, 2); same, except “detrital pools along trail to METS camp; 20–25.iii.2012; SR12-0320-03A” (SEMC, 3); same, except “sandy creek, trail to Kasikasima; flotation; 22.iii.2012; SR12-0322-02A” (SEMC, 7); “04°40.910'N, 56°11.138'W; 78 m; Raleighvallen Nature Reserve, Voltzberg Station; stream margins; 29.vii.2012; leg. Short, Maier, McIntosh, Kadosoe; SR12-0729-02A” (SEMC, 1); same, except “detrital side pool; leg. Short & McIntosh; SR12-0729-02B” (SEMC, 1); same, except “margin of stream; 30.vii.2012; leg. Maier & Kadosoe; SR12-0730-01A” (SEMC, 1); same, except “detrital pools along stream; leg. Short & McIntosh; SR12-0730-01B” (NZCS, SEMC, 10); “4°42.48'N, 56°13.15908'W; 24 m; Raleighvallen Nature Reserve, Lolopaise area; side pool of creek; 14.iii.2016; leg. Short et al.; SR16-0314-02D” (SEMC, 1); “4°40.432'N, 56°11.079'W; 86 m; Raleighvallen Nature Reserve, base of Voltzberg; pooled up stream; 16.iii.2016; SR16-0316-01B” (SEMC, 1); “Raleighvallen Nature Reserve, trail from plateau to Voltzberg stream with roots, mud; 17.iii.2016; leg. J. Girón; SR16-0317-04A” (SEMC, 4); “4°42.48'N, 56°13.15908'W; 24 m; Raleighvallen Nature Reserve, Lolopaise area; intermittent stream margins; flotation; 18.iii.2016; leg. Short et al.; SR16-0318-01D” (SEMC, 2); same, except “intermittent stream pools; pan/screen method; 18.iii.2016; leg. Toussaint et al.; SR16-0318-01E” (SEMC, 1); “Raleighvallen Nature Reserve, Copename River, Voltzberg trail; detrital pools in stream bed; 17.iii.2016; leg. A. Short; SR16-0319-01A” (SEMC, 1); “4°42.48'N, 56°13.15908'W; 24 m; Raleighvallen Nature Reserve, Lolopaise area; intermittent stream pools; 19.iii.2016; leg. Toussaint et al.; SR16-0319-02C” (SEMC, 2); “2°00.397'N, 55°58.371'W; 306 m; Sipaliwini Savanna nature Res., palm swamp nr. 4-Brothers Mts.; mud/detritus; 30.iii.2017; leg. Short & Baca; SR17-0330-03A” (SEMC, 1); same, except “2°00.342'N, 55°58.149'W; 337 m; 4-Brothers Mts.; clearwater stream, sandy with emergent vegetation; 31.iii.2017; SR17-0331-01C” (SEMC, 23); same, except “sandy pools in creek; leg. S. Baca; SR17-0331-01E” (SEMC, 10); same data as holotype (NZCS, SEMC, 23). **VENEZUELA: Amazonas**: “0°50'N, 66°10'W; 140 m; Cerro de la Neblina, 1 Km S Basecamp; along small whitewater stream; pools of dead leaves and sticks; 17.ii.1985; leg. P.J. & P.M. Spangler, R. Faitoute, W. Steiner” (USNM, 2); “Puerto Ayacucho; in small ponds full of dead leaves; 22.i.1985; leg. G.E. Ball” (SEMC, USNM, 5).

##### Differential diagnosis.

*Aulonocharestubulus* can be distinguished by its dark brown general coloration (Fig. 1A–C), and the shape of the aedeagus, which is parallel sided along its basal 2/3, with cylindrical median lobe (Fig. 2E).

##### Description.

Body length 5.8–7.5 mm, width 3.1–4.0 mm. General coloration

dark brown (Fig. 1A–C). Aedeagus (Fig. 2E) with outer margins of parameres subparallel along basal 2/3, slightly concave along apical third; median lobe cylindrical, rounded at apex.

##### Etymology.

Named with the Latin word *tubulus* meaning pipe, after the shape of the median lobe of the aedeagus in this species.

##### Distribution.

Broadly distributed in the Guiana Shield region, from the Orinoco River to central Suriname (Fig. 3).

##### Remarks.

The majority of collecting events of this species are from forested streams, including those actively flowing as well as pooled up, or from isolated marginal pools in the stream bed (Fig. 4B). A few collections were made in forested detrital pools, although most if not all of these were near or associated with riparian corridors. They are usually found in habitats with abundant detritus or decaying organic matter. Females have been observed on numerous occasions to carry their egg case beneath their abdomen.

#### Ephydrolithus gen. nov.

Taxon classificationAnimaliaColeopteraHydrophilidae

http://zoobank.org/2A3C09E9-53A5-4CF8-BE8C-D11D27D363E9

**Figure 5.**
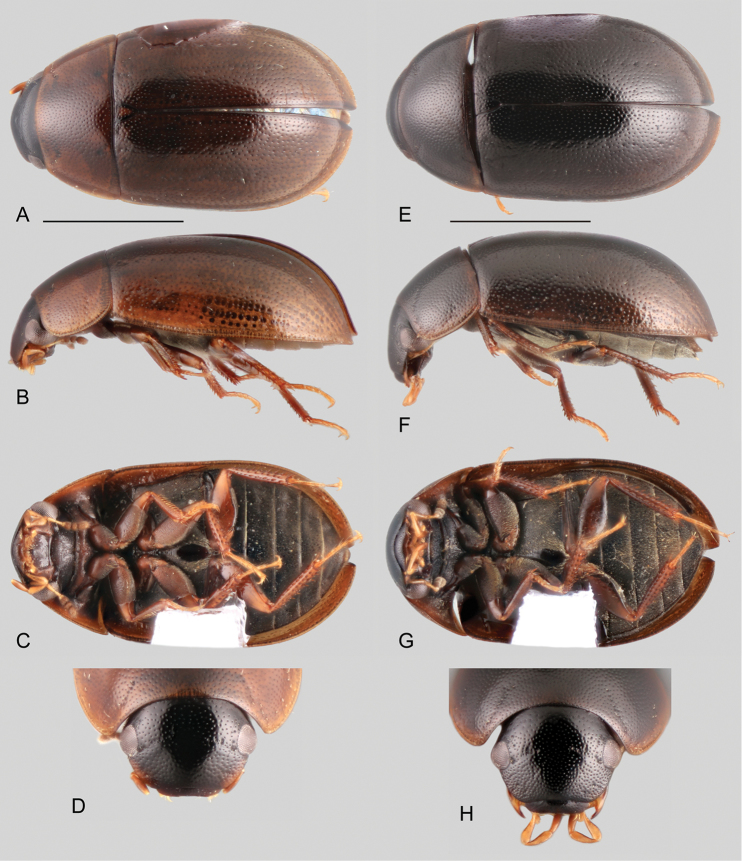
Habitus of *Ephydrolithus* spp.: **A–D***Ephydrolithushamadae*: **A** dorsal view **B** lateral view **C** ventral view **D** head, dorsal view. **E–H***Ephydrolithusteli*: **E** dorsal view **F** lateral view **G** ventral view **H** head, dorsal view. Scale bars: 1 mm.

**Figure 6.**
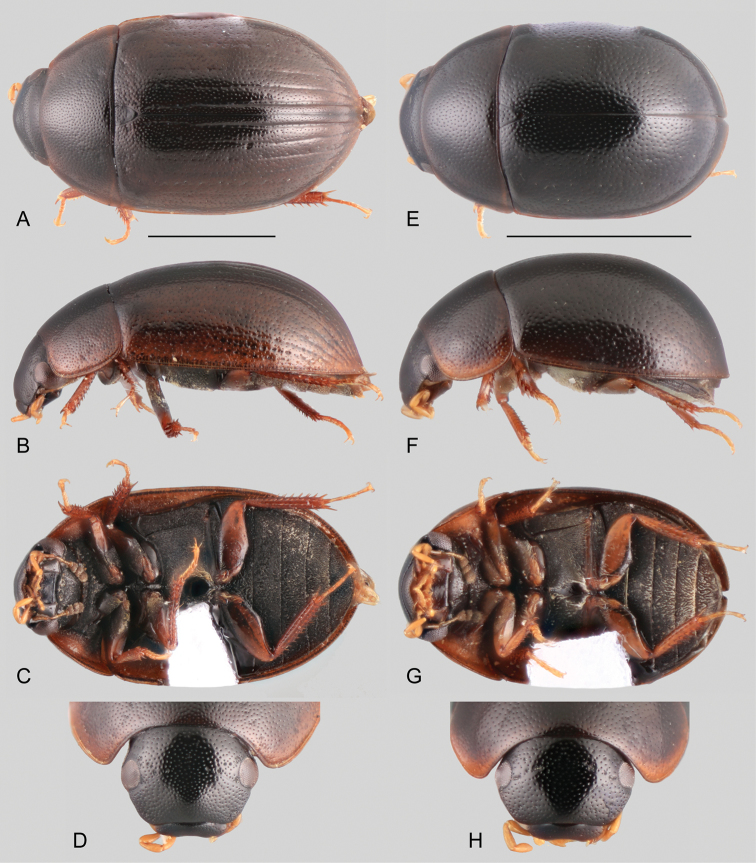
Habitus of *Ephydrolithus* spp.: **A–D***Ephydrolithusogmos*: **A** dorsal view **B** lateral view **C** ventral view **D** head, dorsal view. **E–H***Ephydrolithusminor*: **E** dorsal view **F** lateral view **G** ventral view **H** head, dorsal view. Scale bars: 1 mm.

**Figure 7.**
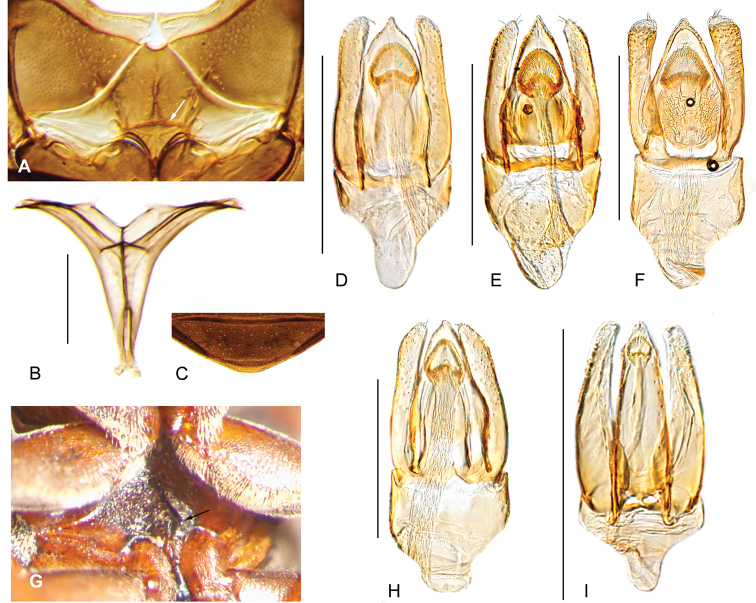
Thorax, abdomen and genitalia of *Ephydrolithus* spp.: **A–D***Ephydrolithushamadae*: **A** ventral view of mesoventrite (white arrow pointing transverse ridge) **B** posterior view of metafurca **C** fifth abdominal ventrite **D** aedeagus **E** aedeagus of *Ephydrolithusteli*. **F, G***Ephydrolithusspiculatus*: **F** aedeagus **G** oblique view of mesoventrite (black arrow pointing well-developed tooth) **H** aedeagus of *Ephydrolithusogmos***I** aedeagus of *Ephydrolithusminor*. Scale bars: 0.25 mm.

**Figure 8.**
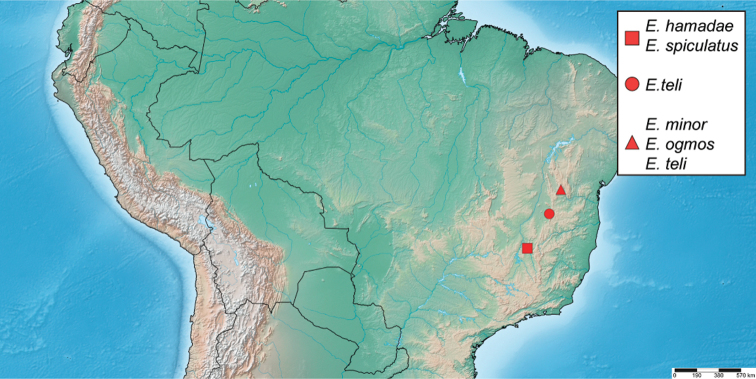
Distribution of *Ephydrolithus* spp.

**Figure 9.**
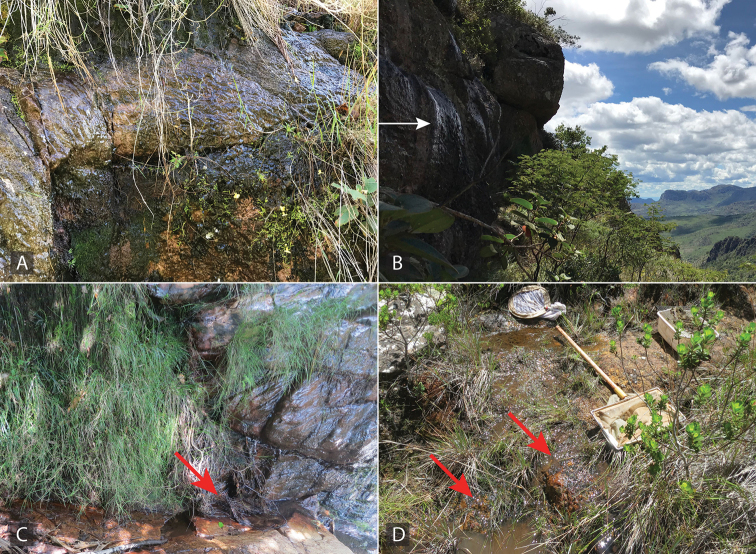
Habitat of *Ephydrolithus* spp. **A, B** habitat and type locality for *E.minor* and *E.ogmos*, Brazil, Pico do Barbado, collecting event BR18-0226-01C **C** habitat and type locality for *E.hamadae* and *E.spiculatus*, Brazil, Cachoeira da Palmeira, collecting event BR18-0302-04A **D** habitat and type locality for E. *teli*, Brazil, Pico do Barbado, collecting event BR18-0226-01B.

Figs 5 , 6 , 7 , 8 , 9

##### Type species.

*Ephydrolithushamadae* sp. nov.

##### Differential diagnosis.

Small beetles (1.8–3.3 mm), oval in dorsal view, moderate to strongly convex in lateral view (e.g., Figs 5B, 6F), yellowish brown to dark brown. Antennae with nine antennomeres (e.g., Fig. 6C). Maxillary palps short (e.g., nearly two thirds the width of the head) and stout (e.g., Fig. 6H). Elytra without sutural striae, and only rarely with impressed striae (e.g., *Ephydrolithusogmos*); ground punctures sharply marked, uniformly and rather densely distributed; systematic punctures slightly larger and deeper than remainder punctures; serial punctures usually absent (present but reduced in *E.ogmos*). Prosternum flat (e.g., Figs 5C, 6C), sometimes only slightly elevated along longitudinal midline. Posterior elevation of mesoventrite usually with a transverse ridge (Fig. 7A; except in *E.ogmos* and *E.spiculatus* which bear a well-developed tooth, e.g. Fig. 6C). Metaventrite densely pubescent, except for a large median teardrop-shaped glabrous patch. Posterior femora glabrous for the most part, with few scattered setae along basal half to basal two thirds, with hydrofuge pubescence along anterodorsal margin; well-developed tibial grooves, sometimes covered by hydrofuge pubescence. Fifth abdominal ventrite apically truncate, with stout setae (e.g., Fig. 7C).

Among Neotropical acidocerines, *Ephydrolithus* has a general resemblance to *Katasophistes* (see Girón and Short 2018), especially by characters of the elytral punctation, which exhibits five rows of deep/large systematic punctures. *Ephydrolithus* can be easily recognized from *Katasophistes* by the mostly glabrous metafemora, with only few scattered setae on anterior surface, as opposed to at most glabrous along apical third in *Katasophistes*. *Ephydrolithus* might also resemble some species of *Chasmogenus*; nevertheless, the absence of sutural striae in *Ephydrolithus* allows its recognition.

*Ephydrolithus* can be distinguished from other Neotropical acidocerines with mostly glabrous metafemora such as *Quadriops* (e.g. Girón and Short 2017) by the entire (as opposed to divided) eyes. From *Tobochares* (e.g. Kohlenberg and Short 2017) *Ephydrolithus* can be distinguished by the number of antennomeres (nine in *Ephydrolithus*, eight in *Tobochares*). In addition, in some species of *Ephydrolithus* the tibial grooves of the metafemora are covered by hydrofuge pubescence, which is an unusual condition among Neotropical acidocerines with mostly glabrous metafemora.

The smaller members of *Ephydrolithus* might resemble species of *Crucisternum* (see Girón and Short 2018), but the prosternal keel of *Crucisternum* easily separates them.

##### Description.

Small beetles, total body length 1.8–3.3 mm; body elongate oval, moderate to strongly convex in lateral view (e.g., Figs 5B, 6F), yellowish brown to dark brown in color, sometimes paler along lateral margins of pronotum and elytra, legs (especially tarsi), mouthparts and antennae. ***Head.*** Frons and clypeus (e.g., Fig. 5H) with moderately marked ground punctures, irregularly and rather densely distributed over the surface, with only few seta-bearing systematic punctures along lateral areas of frons and clypeus; surface between punctures smooth and shiny. Eyes oval in dorsal view, separated by nearly 5 × width of one eye; in lateral view, anterior margin slightly emarginate. Clypeus trapezoid, with medial surface moderately convex, and anterior corners forming widely rounded obtuse angles; anterior margin of clypeus widely roundly emarginate, 0.7 × width of posterior margin; membranous preclypeal area absent. Labrum 0.7 × as wide as anterior margin of clypeus, fully exposed, nearly 1/3 as long, and usually collinear to clypeus (e.g., Fig. 6D); dorsal surface convex, with fine punctures; anterior margin roundly bent inwards, mesally emarginate and with tiny denticles along emargination; anterior corners fringed by setae. Temporae densely covered by very short and fine setae (hydrofuge pubescence). Mentum parallel sided, with surface mostly smooth and undulated, sometimes anteromesally depressed; anterior margin mesally depressed, usually depression marked by a u-shaped transverse carina. Submentum sunken and pubescent at base, glabrous, shiny, and ascending at apex; well-developed ocular ridge. Maxilla (see Fig. 6C) with ventral surface of cardo and stipes smooth and shiny, at most with few scattered and shallow punctures; cardo positioned collinear to oblique to ventral surface of head; outer dorsal margin of palpifer with a row of stiff, decumbent, spiniform setae; limit between cardo and stipes parallel to posterior margin of mentum; maxillary palps curved inward, yellowish, nearly as long as antennae, short (e.g., nearly two thirds the width of the head) and stout (e.g., Fig. 6H); palpomere 1 strongly widened near apex (with outer apical margin strongly convex); palpomere 2 gradually widening towards apex; palpomere 3 fusiform, bearing apical sensilla; all palpomeres similar in length. Mandibles with apex bifid (examined in *E.ogmos*). Labial palps yellow, slightly shorter than mentum, dorsoventrally flattened; palpomere 2 with outer margin strongly convex apicad of midpoint, sometimes with one preapical seta on outer surface; palpomere 3 digitiform, with a long subapical seta on outer corner. Antennae (see Fig. 6C) with nine antennomeres, usually yellow with darker club; antennomere 1 with surface evenly convex near base, reaching midpoint of ventral surface of eye (reaching cardo-stipes joint), 1.5–2.5 × longer than antennomere 2; antennomere 2 nearly as long as antennomeres 3–5 combined; antennomere 6 forming a well differentiated, asymmetric cupule; antennomeres 7–9 each wider than long, slightly flattened, forming a loosely articulated, pubescent club (antennomere 8 shortest, 9 longest); pubescence of antennomere 9 with few scattered longer setae on apical area. ***Thorax.*** Pronotum widest at base, narrowed anteriorly, surface evenly convex; ground punctation moderate, uniformly dense, with surface between punctures smooth and shiny; seta-bearing systematic punctures forming paired anterolateral semicircles, and paired short posterolateral transverse bands. Scutellar shield of moderate size, triangular, nearly as long as wide, with punctation as in pronotum. Prosternum flat (e.g., Figs 5C, 6C), sometimes only slightly elevated along longitudinal midline (e.g., Fig. 5G), nearly as long as half the length of a procoxa; anterior margin of prosternum straight to slightly convex; surface finely crenulate, with scattered fine setae, slightly impressed along procoxal area; intercoxal process projected from posterior margin of procoxal cavities, rectangularly shaped in outline, mesally longitudinally carinate. Mesoventrite (Fig. 7A, G) not fused to mesepisterna, with anterior margin 0.2–0.4 × as wide as anterior margin of mesepisternum; anterior rib of mesoventrite with median, triangular, pale macula; posterior elevation of mesoventrite either with a sharp, low, transverse, curved ridge (Figs 5C, 7A), or bearing a basally transverse, well-developed tooth that extends anteriorly as a longitudinal carina (Figs 6C, 7G); surface of mesoventrite with posterolateral smooth and glabrous areas; mesepisternum obliquely widely concave; mesepimeron trapezoid, with pubescent surface. Mesofurca (examined in *E.hamadae*) with short arms, 0.9 × length of mesocoxae; apical half of arms free, somewhat triangular at apex. Metaventrite posteromesally elevated, with elevation somewhat narrow anteriorly, widening posteriorly; surface of metaventrite densely pubescent, except for a median to posteromedian, large teardrop-shaped glabrous patch; anteromedian area of metaventrite with a deep and narrow transverse depression before anterior intercoxal process. Metepisterna nearly 4 × longer than wide, slightly narrowing at posterior end. Metepimeron triangular and posteriorly slightly projected. Metafurca (examined in *E.hamadae*, Fig. 7B) 1.3 × wider than long, with furcal arms 0.8 × the length of stalk; stalk triangular (wider near the crux, gradually narrowing ventrally), with paired longitudinal keels extending along basal third of posterior face, fusing together towards crux, with a well-developed median keel on anterior face extending to anterior margin of dorsal sheets; outer margins of stalk diverging from basal third towards crux; furcal arms somewhat trapezoid, with apex (hemiductus) roundly explanate, with apex pointing laterally; anterior tendons inserted at basal third of dorsal edge of furcal arms; well-developed dorsal sheaths, wider than widest point of lateral sheaths. ***Elytra.*** Surface even (without elevations or depressions), without sutural striae (in *E.ogmos* elytral striae well-marked, more strongly so along stria 1); ground punctures sharply marked, uniformly and rather densely distributed; seta-bearing systematic punctures rather enlarged and deep, forming five longitudinal rows along each elytron, fifth row very close to outer margin of elytron; serial punctures usually absent (present but reduced in *E.ogmos*); elytral margins slightly flared. Epipleura well developed, surface rather oblique, with fine setae, anteriorly wide, gradually narrowing posteriorly, extending up to line of posterior margin of metaventrite; inner margin of epipleura slightly concave at articulation of anterior outer corner of metepisternum; well-developed pseudepipleura, rather obliquely positioned, anteriorly nearly as wide as anterior portion of epipleura, narrowing towards line of posterior margin of metaventrite, extending as narrow band along remainder outer margin of elytron. Hind wings well developed (examined in *E.hamadae* and *E.teli*). ***Legs.*** Pro- and mesofemora covered with hydrofuge pubescence along at least basal half; metafemora with hydrofuge pubescence as a narrow stripe along basal 2/3 of anterodorsal margin, remainder anterior surface usually smooth and shiny, with only few scattered setae; all femora antero-posteriorly flattened, with sharp tibial grooves; sometimes tibial grooves with hydrofuge pubescence (in *E.hamadae* and *E.teli*). Tibiae slender, weakly flattened, with well-developed spines; protibiae with a median longitudinal row of long setae along anterior surface; apical spurs of protibiae rather large and slender. All tarsi with five tarsomeres, bearing long apical hair-like setae on dorsal face, and two lateral rows of hair-like spines on ventral face of tarsomeres 2–4; pro- and mesotarsomeres 1–4 similar in size and shape; pro- and mesotarsomere 5 similar in size to pro- and mesotarsomeres 1–4 combined; metatarsomere 2 nearly as long as tarsomeres 3–4 combined; metatarsomere 5 similar in size to metatarsomere 2, without spines on ventral face; claws rather large, curved; well-developed empodium, bearing a pair of long, curved apical setae. ***Abdomen.*** Abdomen with five ventrites, very weakly convex medially; all ventrites with uniform, dense, fine pubescence; posterior margin of fifth ventrite truncate, set with a row of thick, flat spine-like setae (Fig. 7C). Aedeagus (Fig. 7D–F, H, I) with outer margins convex, straight or sinuate, with basal piece between 0.45 and 0.9 X the length of parameres; median lobe somewhat triangular in shape, with well-developed lateral basal apodemes; widest point of median lobe wider than widest point of each paramere; apex of median lobe widely to narrowly acute, sometimes “pinched” (e.g. *E.hamadae*, Fig. 7D); parameres nearly as long as median lobe, with greatest width near base, bearing apical setae; well-developed gonopore, preapically situated.

##### Larvae.

The immature stages are unknown.

##### Etymology.

Named by the combination of the Greek words *ephydros* meaning wet, and *lithus* meaning rock, in reference to the seepage habitat in which the genus has been collected. To be treated as neuter.

##### Distribution.

The genus is currently only known from the northeastern highlands of Brazil (Bahía, Minas Gerais) on the Brazilian Shield (Fig. 8).

##### Remarks.

Species of *Ephydrolithus* have been collected in an altitudinal range between 568 and 1705 m. All known species are exclusively associated with rock seepages (see Fig. 9).

### Characters of taxonomic importance for *Ephydrolithus*

Even though members of *Ephydrolithus* are externally relatively homogeneous, there are some useful characters for species identification.

**Body size.** Most *Ephydrolithus* species range in size from 2.6 to 3.3 mm. *Ephydrolithusminor* is the smallest species, with body size ranging from 1.8 to 2.2 mm.

**Elytral surface.** Most species of *Ephydrolithus* lack elytral striae; only *E.ogmos* has impressed striae along almost the entire length of the elytra.

**Tibial grooves of metafemora.** In some species of *Ephydrolithus* the tibial grooves of the metafemora are covered by hydrofuge pubescence. Only *E.minor* and *E.ogmos* have glabrous metafemoral tibial grooves.

**Posterior elevation of mesoventrite.** Usually the elevation bears a sharp, low, transverse, curved ridge (Figs 5C, 7A). Only *E.ogmos* and *E.spiculatus* bear a pointed spine (Figs 6C, 7G).

**Aedeagus.** The overall forms and proportions of the aedeagus of *Ephydrolithus* species are very similar among species, except for *E.minor*, which has a comparatively shorter basal piece and narrower median lobe (see Fig. 7I).

### Key to the species of *Ephydrolithus*

**Table:** 

1	Elytra with well-defined and impressed striae (Fig. 6A, B)	*** E. ogmos ***
–	Elytral without impressed striae (Figs 5A, E, 6E)	**2**
2	Body strongly convex, 1.8–2.2 mm in length; anterior surface of pro- and mesofemora covered by hydrofuge pubescence along basal half (Fig. 6G)	*** E. minor ***
–	Body moderately convex, 2.4–2.9 mm in length; anterior surface of pro- and mesofemora covered by hydrofuge pubescence along basal two thirds (Fig. 5C, G)	**3**
3	Posterior elevation of mesoventrite bearing a pointed spine (Fig. 7G)	*** E. spiculatus ***
–	Posterior elevation of mesoventrite with a sharp, low, transverse, curved ridge (Figs 5C, 7A)	**4**
4	Parameres of aedeagus 1.2–1.3 × longer than basal piece; median lobe 2 × longer than its greatest width; outer margins of apex of median lobe straight to slightly sinuate (apex of median lobe triangular) (Fig. 7E)	*** E. teli ***
–	Parameres of aedeagus 1.5–1.6 × longer than basal piece; median lobe nearly 2.5 × longer than its greatest width; outer margins of apex of median lobe clearly sinuate (apex of median lobe “pinched”) (Fig. 7D)	*** E. hamadae ***

#### Ephydrolithus hamadae sp. nov.

Taxon classificationAnimaliaColeopteraHydrophilidae

http://zoobank.org/3129CD1D-7BAE-4C2B-86B4-25B08D27E712

Figs 5A–D , 7A–D , 8 , 9C

##### Type material.

**Holotype** (♂): “BRAZIL: Minas Gerais: Lassance; 17.83384S, 44.50515W; 568 m; Cachoeira da Palmeira; flotation of root mats and moss from side of waterfall & seepage; 2.iii.2018; leg. Benetti & team; BR18-0302-04A” (INPA). **Paratypes (7): BRAZIL: Minas Gerais**: Same data as holotype (INPA, SEMC, 7 including DNA voucher SLE 1506).

##### Differential diagnosis.

*Ephydrolithushamadae* is very similar to *E.teli*. Both species can only be distinguished from each other by characteristics of the aedeagus.

##### Description.

Body length 2.6–3.2 mm, width 1.5–1.7 mm. Body elongate oval, moderately convex (Fig. 5B). General coloration yellowish to dark brown, slightly paler along margins of pronotum and elytra. Posterior elevation of mesoventrite with well-defined, curved transverse ridge. Elytra without striae or serial punctures. Pro- and mesofemora covered with hydrofuge pubescence along basal 2/3; metafemora with hydrofuge pubescence on tibial grooves. Apex of fifth abdominal ventrite truncate (Fig. 7C). Aedeagus (Fig. 7D) with basal piece 0.6 × the length of parameres; parameres nearly 0.5 × greatest width of median lobe, with outer margins slightly sinuate; apex of parameres rounded, obliquely directed; apex of median lobe “pinched”, narrowly rounded.

##### Etymology.

Named after Neusa Hamada from the Instituto Nacional de Pesquisas da Amazônia, Manaus (INPA), in recognition of her support on recent expeditions collecting aquatic beetles in Brazil.

##### Distribution.

Known only from the type locality (Fig. 8).

##### Remarks.

This species was collected by gathering moss and roots from bottom and margin of a seepage that was next to a large waterfall (Fig. 9C). Specimens were collected by placing the moss and roots in a pan with water, where they floated to the surface along with one specimen of *E.spiculatus*.

#### Ephydrolithus minor sp. nov.

Taxon classificationAnimaliaColeopteraHydrophilidae

http://zoobank.org/E49E34BB-6106-451D-A670-0ACF02FBB604

Figs 6E–H , 7I , 8 , 9A, B

##### Type material.

**Holotype** (♂): “BRAZIL: Bahia: Abaíra; 13.29053S, 41.90489W; 1705 m; Pico do Barbado W of Catolés; vertical seep on rock; 26.ii.2018; leg. Benetti & team; BR18-0226-01C” (INPA). **Paratypes (15): BRAZIL: Bahia**: Same data as holotype (SEMC, 8 including DNA vouchers SLE-1511, SLE-1512; INPA, 7).

##### Differential diagnosis.

*Ephydrolithusminor* is easily recognized among its congeners by its small size.

##### Description.

Body length 1.8–2.2 mm, width 0.9–1.3 mm. Body elongate oval, strongly convex (Fig. 6F). General coloration dark brown, slightly paler along margins of pronotum. Posterior elevation of mesoventrite with well-defined, curved transverse ridge. Elytra without striae or serial punctures. Pro- and mesofemora covered with hydrofuge pubescence along basal half; metafemora with glabrous tibial grooves. Apex of fifth abdominal ventrite rounded. Aedeagus (Fig. 7I) with basal piece 0.45 × the length of parameres; greatest width of parameres similar to greatest width of median lobe, with outer margins evenly convex; apex of parameres truncate, obliquely directed; apex of median lobe rather widely rounded.

##### Etymology.

Named with the Latin word *minor* meaning small, in reference to the species being the smallest member of the genus.

##### Distribution.

Only known from the type locality, Pico do Barbado (Fig. 8).

##### Remarks.

The type series was collected on a high-elevation seepage over a vertical cliff. The rock face had moss and algal growth on same areas (Fig. 9A, B).

#### Ephydrolithus ogmos sp. nov.

Taxon classificationAnimaliaColeopteraHydrophilidae

http://zoobank.org/7ECC48CA-7772-4FD1-B5EE-820957C3B5C3

Figs 6A–D , 7H , 8 , 9A, B

##### Type material.

**Holotype** (♂): “BRAZIL: Bahia: Abaíra; 13.29053S, 41.90489W; 1705 m; Pico do Barbado, W of Catolés; vertical seep on rock; 26.ii.2018; leg. Benetti & team; BR18-0226-01C” (INPA). **Paratypes (4): BRAZIL: Bahia**: Same data as holotype (SEMC, 2 including DNA voucher SLE-1510; INPA, 2).

##### Differential diagnosis.

*Ephydrolithusogmos* is easily distinguished from all the other known species by its well-defined striae along the posterior third of the elytra.

##### Description.

Body length 3.1–3.3 mm, width 1.8–2.0 mm. Body elongate oval, strongly convex (Fig. 6B). General coloration brown, slightly paler along margins of pronotum and elytra. Posterior elevation of mesoventrite with well-developed spine, forming high anterior carina. Elytra with well-developed striae along posterior half and reduced serial punctures. Pro- and mesofemora covered with hydrofuge pubescence along basal half; metafemora with glabrous tibial grooves. Apex of fifth abdominal ventrite truncate. Aedeagus (Fig. 7H) with basal piece 0.7 × the length of parameres; parameres nearly 0.7 × greatest width of median lobe, with outer margins slightly sinuate; apex of parameres rounded, obliquely directed; apex of median lobe widely acute.

##### Etymology.

Named with the Greek word *ogmos* meaning furrow, in reference to the well-defined elytral striae of the species.

##### Distribution.

Only known from the type locality, Pico do Barbado (Fig. 8).

##### Life history.

The type series was collected on a high-elevation seepage over a vertical cliff. The rock face had moss and algal growth on same areas (Figs 9A, B).

#### Ephydrolithus spiculatus sp. nov.

Taxon classificationAnimaliaColeopteraHydrophilidae

http://zoobank.org/EEAFB6BE-5C09-4572-B963-321840A6E871

Figs 7F, G , 8 , 9C

##### Type material.

**Holotype** (♂): “BRAZIL: Minas Gerais: Lassance; 17.83384S, 44.50515W; 568 m; Cachoeira da Palmeira; flotation of root mats and moss from side of waterfall & seepage; 2.iii.2018; leg. Benetti & team; BR18-0302-04A” (INPA).

##### Differential diagnosis.

*Ephydrolithusspiculatus* is very similar to *E.hamadae* and *E.teli*. It can be easily distinguished from both by the presence of a pointed spine on the posterior elevation of the mesoventrite (see Fig. 7G).

##### Description.

Body length 3.2 mm, width 1.7 mm. Body elongate oval, moderately convex. General coloration brown, slightly paler on pronotum and along margins of elytra, with dark brown head. Posterior elevation of mesoventrite with a pointed spine (Fig. 7G). Elytra without striae or serial punctures. Pro- and mesofemora covered with hydrofuge pubescence along basal 2/3; metafemora with hydrofuge pubescence along basal 2/3 of anterior margin, and on tibial grooves. Apex of fifth abdominal ventrite truncate. Aedeagus (Fig. 7F) with basal piece 0.9 × the length of parameres; parameres nearly 0.3 × greatest width of median lobe, with outer margins nearly straight for most of their length; apex of parameres truncate, with outer corners broadly rounded; apex of median lobe widely acute.

##### Etymology.

Named with the Latin word *spiculatus* meaning sharpen to a point, in reference to the pointed spine on the posterior elevation of the mesoventrite.

##### Distribution.

Known only from the type locality (Fig. 8).

##### Remarks.

This species was collected by gathering moss and roots from bottom and margin of a seepage that was next to a large waterfall (Fig. 9C). The only known specimen was collected by placing the moss and roots in a pan of water, where it floated to the surface along with several specimens of *E.hamadae*.

#### Ephydrolithus teli sp. nov.

Taxon classificationAnimaliaColeopteraHydrophilidae

http://zoobank.org/A1E4159C-2BAC-4B4D-B215-2D4A109C1D5E

Figs 5E–H , 7E , 8 , 9D

##### Type material.

**Holotype** (♂): “BRAZIL: Bahia: Abaíra; 13.29053S, 41.90489W; 1705 m; Pico do Barbado, W of Catolés; flotation of mud and moss from seepage; 26.ii.2018; leg. Benetti & team; BR18-0226-01B” (INPA). **Paratypes (8): BRAZIL: Bahia**: Same data as holotype (SEMC, 6 including DNA voucher SLE-1486). **Minas Gerais**: “Monte Azul; 15.17067S, 42.80351W; 970 m; Serra do Espinhaço, c. 7 Km E of Monte Azul; seepage areas in stream on rock; 28.ii.2018; leg. Benetti & team; BR18-0228-02B” (SEMC, 3 including DNA voucher SLE-1509).

##### Differential diagnosis.

*Ephydrolithusteli* is very similar to *E.hamadae*. Both species can only be distinguished from each other by characteristics of the aedeagus.

##### Description.

Body length 2.8–3.3 mm, width 1.5–1.9 mm. Body elongate oval, moderately convex (Fig. 5F). General coloration dark brown. Posterior elevation of mesoventrite with well-defined, curved transverse ridge. Elytra without striae or serial punctures. Pro- and mesofemora covered with hydrofuge pubescence along basal 2/3; metafemora with hydrofuge pubescence on tibial grooves. Apex of fifth abdominal ventrite truncate. Aedeagus (Fig. 7E) with basal piece 0.85 × the length of parameres; parameres nearly 0.4 × greatest width of median lobe, with outer margins only slightly convex; apex of parameres truncate, obliquely directed; apex of median lobe triangular, very narrowly rounded.

##### Etymology.

Named with the Latin word *teli* meaning spear, in reference to the shape of the median lobe of the aedeagus of the species.

##### Distribution.

Known from two localities in the highlands of northeastern Brazil (Fig. 8).

##### Remarks.

Both collections of this species were taken from rocky seepage habitats (e.g., Fig. 9D).

#### Primocerus gen. nov.

Taxon classificationAnimaliaColeopteraHydrophilidae

http://zoobank.org/0EA5176F-B2BB-4E50-8799-C93F09B412B6

**Figure 10.**
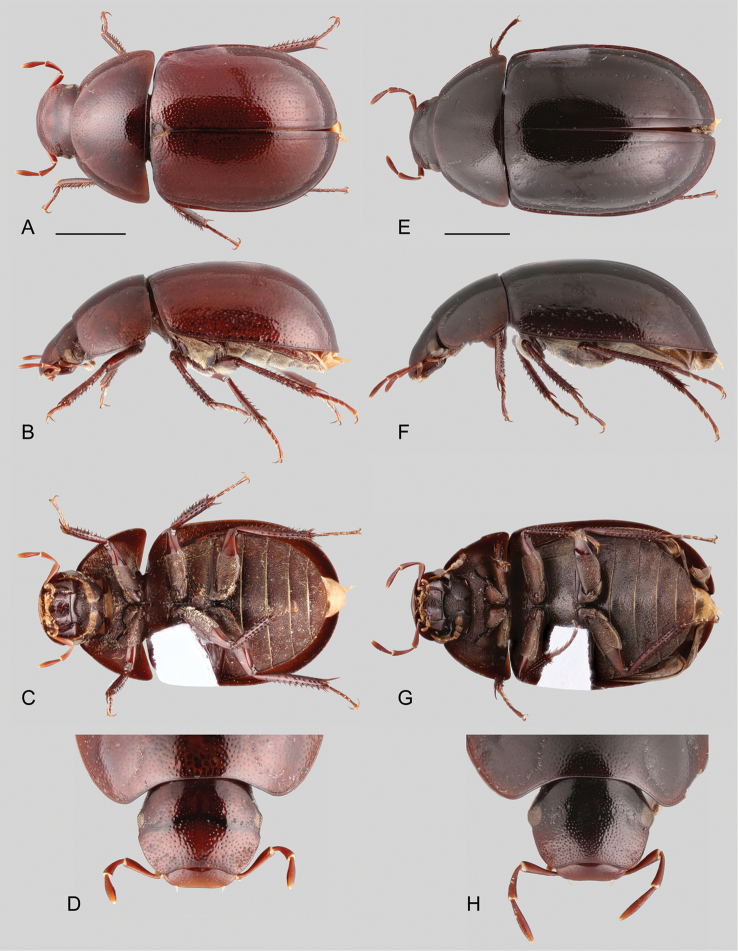
Habitus of *Primocerus* spp.: **A–D***Primocerusocellatus*: **A** dorsal view **B** lateral view **C** ventral view **D** head, dorsal view. **E–H***Primocerusgigas*: **E** dorsal view **F** lateral view **G** ventral view **H** head, dorsal view. Scale bars: 1 mm.

**Figure 11.**
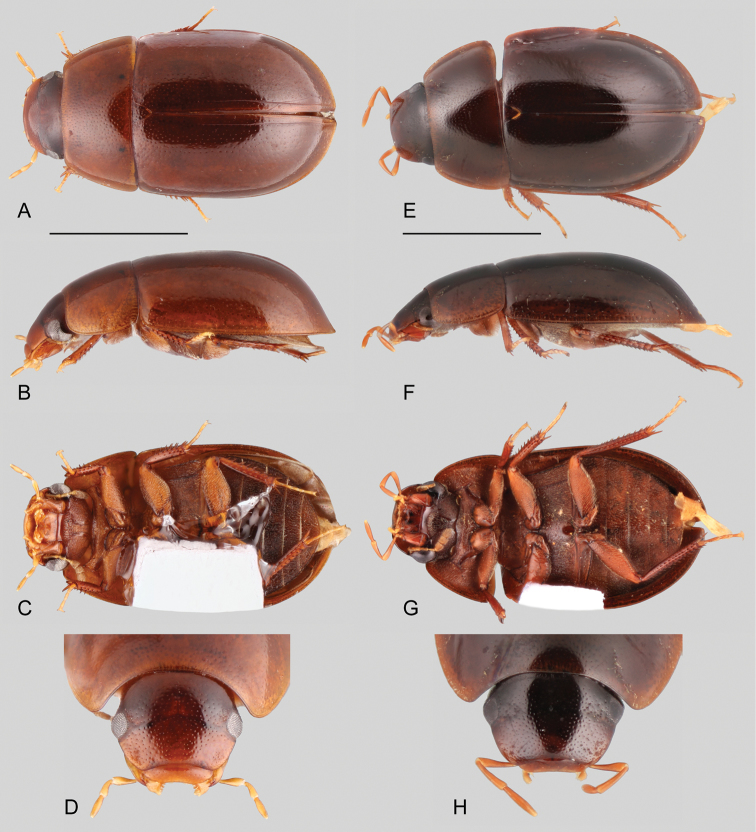
Habitus of *Primocerus* spp.: **A–D***Primoceruscuspidis*: **A** dorsal view **B** lateral view **C** ventral view **D** head, dorsal view. **E–H***Primocerusneutrum*: **E** dorsal view **F** lateral view **G** ventral view **H** head, dorsal view. Scale bars: 1 mm.

**Figure 12.**
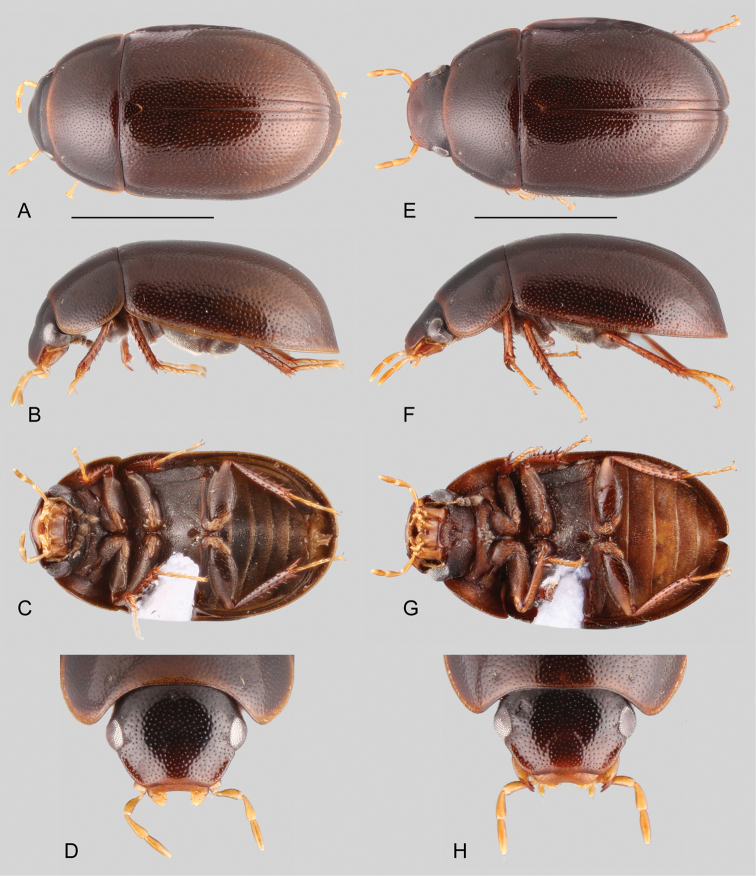
Habitus of *Primocerus* spp.: **A–D***Primocerusmaipure*: **A** dorsal view **B** lateral view **C** ventral view **D** head, dorsal view. **E–H***Primoceruspijiguaense*: **E** dorsal view **F** lateral view **G** ventral view **H** head, dorsal view. Scale bars: 1 mm.

**Figure 13.**
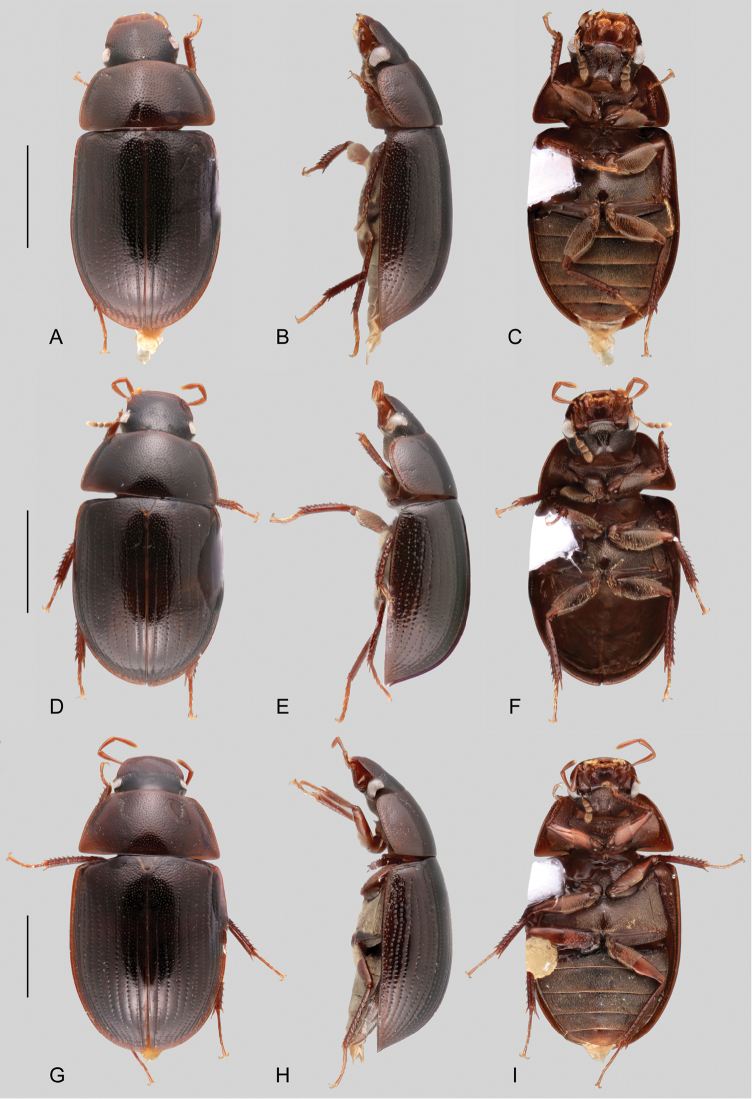
Habitus of *Primocerus* spp.: **A–C***Primoceruspetilus*: **A** dorsal view **B** lateral view **C** ventral view. **D–F***Primocerusstriatolatus*: **D** dorsal view **E** lateral view **F** ventral view. **G–I***Primocerussemipubescens*: **G** dorsal view **H** lateral view **I** ventral view. Scale bars: 1 mm.

**Figure 14.**
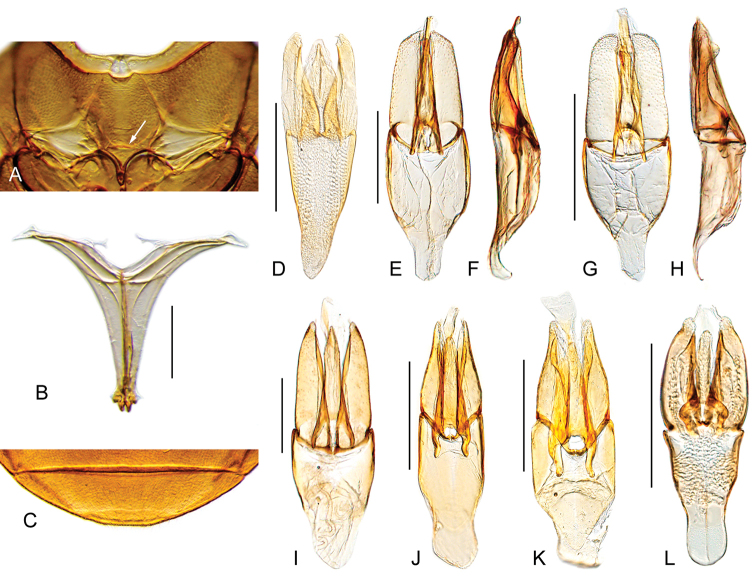
Thorax, abdomen and aedeagus of *Primocerus* spp.: **A–D***Primocerusneutrum*: **A** ventral view of mesoventrite (white arrow pointing transverse ridge) **B** posterior view of metafurca **C** fifth abdominal ventrite **D** aedeagus. **E–L** aedeagus: **E, F***Primocerusmaipure*: **E** dorsal view **F** lateral view. **G, H***Primoceruspijiguaense*: **G** dorsal view **H** lateral view **I***Primocerusgigas***J***Primoceruspetilus***K***Primocerusstriatolatus***L***Primoceruscuspidis.* Scale bars: 0.25 mm.

**Figure 15.**
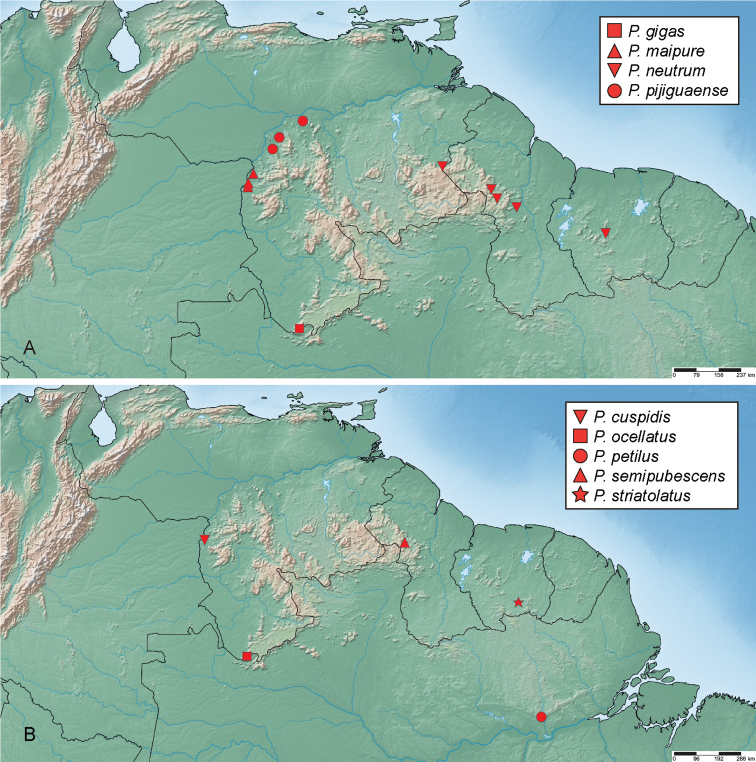
Distribution of *Primocerus* spp.

**Figure 16.**
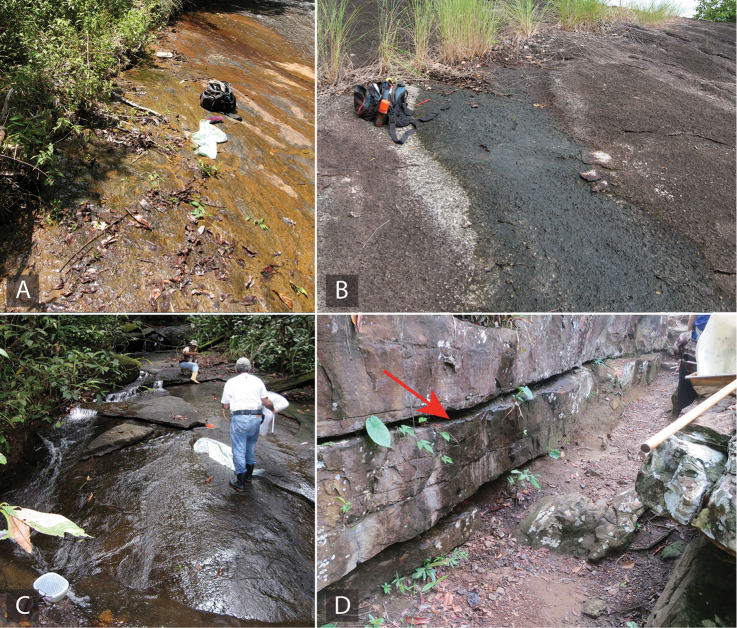
Habitat of *Primocerus* spp. **A** habitat and type locality for *P.cuspidis*, Venezuela, Tobogán de la Selva, collecting event AS-08-080b **B** habitat and type locality for *P.pijiguaense*, Venezuela, Los Pijiguaos, collecting event AS-07-015 **C** habitat and type locality for *P.neutrum*, Venezuela, along La Escalera, collecting event AS-08-058 **D** habitat and type locality for *P.petilus*, Brazil, Vale do Paraiso, collecting event BR18-0203-01G.

Figs 10 , 11 , 12 , 13 , 14 , 15 , 16

##### Type species.

*Primocerusneutrum* sp. nov.

##### Differential diagnosis.

Small to medium sized beetles (2.4–4.9 mm), elongate oval in dorsal view, moderate to strongly convex in lateral view (e.g., Figs 11F, 12B), brown, dark brown, reddish brown, or rather orange. Antennae with eight antennomeres (e.g., Fig. 10G). Maxillary palps short to moderately long (e.g., shorter to nearly as long as the width of the head; e.g., Figs 10H, 12H). Elytra with sutural striae; elytral punctures from shallow to sharply marked (e.g., Figs 11E, 12E); serial punctures, ground punctures and systematic punctures similar in size and degree of impression throughout elytra; all punctures seemingly arranged in rows, sometimes evidently so. Prosternum flat to mesally only slightly produced. Posterior elevation of mesoventrite with a curved transverse ridge, rather sharp and low (Fig. 14A), except in *P.cuspidis* which bears a sharp, pyramidal (triangular) projection. Posteromesal glabrous patch on metaventrite nearly as wide as long. Pubescence on anterior surface of metafemora ranging from sparse to densely covering basal three fourths of the femur (e.g., Figs 12C, 10G, 11G). Fifth abdominal ventrite apically rounded, truncate or slightly emarginate, usually with stout setae (e.g., Fig. 14C). Basal piece of aedeagus as long as or longer than parameres; median lobe triangular, nearly as wide at base as basal width of one paramere, with apical projection (Fig. 14D–L); gonopore absent (Fig. 14D–L).

At first sight, the dorsally smoother members of *Primocerus* (Figs 10, 11) can be mistaken for *Chasmogenus*, given that both genera exhibit sutural striae. The presence of a transverse curved ridge (sometimes very low) on the posterior elevation of the mesoventrite distinguishes *Primocerus* from *Chasmogenus*, in which the mesoventrite is either flat, broadly elevated or with a longitudinal elevation (e.g., figs 2, 4 in Clarkson and Ferreira 2014). In addition, the maxillary palps of most *Chasmogenus* species are nearly 1.5 × longer than the maximum width of the head, whereas in *Primocerus* the maxillary palps are nearly as long as the width of the head.

Punctate members of *Primocerus* (in particular *P.maipure* and *P.pijiguaense*, Fig. 12) may resemble some species of *Tobochares* (e.g. *T.canthus*, *T.pallidus*; Kohenberg and Short 2017); striate *Primocerus* may resemble a very small *Radicitus* (see Short and García 2014). In those cases, *Primocerus* can be easily recognized by the presence of sutural striae. Some *Primocerus* may also superficially resemble certain New World cylomine genera, such as *Andotypus* (see Fikáček et al. 2014), from which it may be distinguished by the fully exposed labrum of *Primocerus*.

In addition, the presence of sutural striae and the relative size of the basal piece of the aedeagus resemble some species of *Enochrus* (Enochrinae) in that the basal piece is as long as or longer than the median lobe + parameres (e.g. see figs 11 and 14 in Fernández 2006). The maxillary palps curved inwards in *Primocerus* (as opposed to zig-zag-like as in *Enochrus*) allows for its recognition.

The aedeagus of *Primocerus* is so far unique among the Acidocerinae in the lack of a well-developed gonopore, and the presence of a lightly sclerotized projection beyond the apex of the median lobe.

##### Description.

Small to medium sized beetles, total body length 2.4–4.9 mm; body elongate oval, moderate to strongly convex in lateral view (e.g., Figs 11F, 12B); orange brown (Fig. 11A–D), reddish brown (Fig. 10 A–D), to dark brown in coloration (e.g., Fig. 13), usually uniform along body regions, sometimes slightly paler along margins, pronotum, ventral surfaces, and appendages, particularly maxillary palps and tarsi. ***Head.*** Frons and clypeus with either shallow (e.g., Fig. 10D) or sharply marked (e.g., Fig. 12D) ground punctures, irregularly and rather densely distributed over the surface, accompanied by scattered seta-bearing systematic punctures, particularly noticeable along anterior and inner margins of eyes, and lateral areas of clypeus; surface between punctures smooth and shiny. Clypeus roughly trapezoid, with posterior margin wider than anterior margin; anterior corners roundly angulated, anterior margin widely roundly emarginate; membranous preclypeal area not visible (visible in *Chasmogenus*; e.g., fig. 28 in Clarkson and Ferreira 2014); surface mesally moderately convex, laterally flattened to slightly concave (Figs 12D, H). Eyes subquadrate in dorsal view, usually protruding from outer outline of head. Labrum wide, fully exposed, collinear to perpendicular to clypeus, and usually around 0.3 times as long as clypeus (e.g., Fig. 10D); dorsal surface flat to convex, with scattered fine punctures; anterior margin markedly roundly bent inwards, mesally emarginate, with tiny denticles along emargination, and setae on lateral areas of anterior margin. Temporae densely covered by very short and fine setae (hydrofuge pubescence). Mentum parallel sided, often with lateral margins densely fringed by short setae; surface rather flat, smooth, and glabrous, sometimes with lateral oblique longitudinal ridges, and few crenulations; anterior margin with wide, deep, concave median impression, sometimes marked by a transverse carina. Submentum sunken, concave, and pubescent at base, glabrous, shiny, flat and ascending at apex; ocular ridge of variable development. Maxilla (e.g., Fig. 10G) with ventral surface of cardo and stipes smooth, shiny, and glabrous; outer dorsal margin of palpifer with a row of stiff, decumbent, spiniform setae; limit between cardo and stipes oblique; maxillary palps curved inward, brown to orange or yellow, longer than antennae, short to moderately long (e.g., shorter to nearly as long as the width of the head; e.g., Figs 10H, 12H); maxillary palpomere 1 gradually broadening towards apex, with inner margin straight and outer margin apically convex; apex of palpomere 3 bearing sensilla; palpomeres 1 and 3 similar in length, palpomere 2 only slightly shorter. Mandibles with apex bifid (observed in *P.gigas*, *P.pijiguaense*, *P.striatolatus* and *P.petilus*; e.g., Fig. 12H). Labial palps yellowish to brown, usually nearly as long as mentum, dorsoventrally flattened; palpomere 2 with outer margin convex apicad of midpoint, sometimes with setae near apex; palpomere 3 digitiform to somewhat kidney-shaped, with one or two long subapical setae on outer margin. Antennae (e.g., Fig. 10G) with eight antennomeres, slightly paler than general coloration of head; antennomere 1 anteriorly projected near base, at most reaching midpoint of ventral surface of eye, reaching to surpassing cardo-stipes joint, nearly 2.0 × longer than antennomere 2; antennomere 2 nearly as long as antennomeres 3–4 combined; antennomere 5 forming a well differentiated, symmetric cupule; antennomeres 6–8 slightly flattened, forming a loosely articulated, pubescent club (antennomere 7 shortest, 8 longest); apex of antennomere 8 with longer setae than general pubescence of club. ***Thorax.*** Pronotum widest at base, narrowed anteriorly, surface evenly convex; anterior and posterior corners widely rounded, sometimes posterior corners almost forming a sharp straight angle (e.g., Fig. 10G); anterior and posterior margins nearly straight; ground punctation either shallow or sharp, uniformly dense, with surface between punctures smooth and shiny; seta-bearing systematic punctures forming paired anterolateral semicircles. Scutellar shield of moderate size, triangular, nearly as long as wide, with punctation as in pronotum. Prosternum nearly as long as 0.7 × the length of a procoxa; anterior margin of prosternum mesally projected as a wide triangle, apically either acute or rounded (except in *P.ocellatus*); surface of prosternum flat to only weakly broadly convex, covered by scattered, fine, rather long setae; intercoxal process projected from posterior margin of procoxal cavities, rectangular in outline, mesally longitudinally carinate. Mesoventrite (Fig. 14A) not fused to mesepisterna, with anterior margin nearly 0.3 × as wide as anterior margin of mesepisternum; anterior rib of mesoventrite bearing paired medial teardrop-shaped, pearlescent maculae; posterior elevation of mesoventrite with a transverse curved ridge, rather sharp and low, reduced in *P.maipure*, *P.pijiguaense* and *P.ocellatus*, with a sharp, pyramidal (triangular) spine-like projection in *P.cuspidis* (Fig. 11C); surface of mesoventrite reticulated for the most part, covered by scattered, fine and rather long setae, with anteromedial depression, and posterolateral smooth and glabrous areas; mesepisternum obliquely widely concave, with reticulated surface; mesepimeron trapezoid, with reticulate and pubescent surface. Mesofurca (examined in *P.neutrum*) with short arms, 0.75 × length of mesocoxae; apical half of arms free, explanate at apex, somewhat square. Metaventrite mesally widely elevated, rather wide throughout and flat posteromesally; surface densely pubescent, except for posteromesal nearly as wide as long glabrous patch, and soemtimes postero-lateral areas (Fig. 10G; except in *P.ocellatus*, Fig. 10D). Metepisterna 3–4 × longer than wide, narrowing only at posterior end. Metepimeron clearly visible, triangular. Metafurca (examined in *P.neutrum*, Fig. 14B) 1.3 × wider than long, with furcal arms slightly shorter than stalk; stalk triangular (wider near the crux, gradually narrowing ventrally), with paired longitudinal keels extending along basal third of posterior face, fusing together towards crux, with a well-developed median keel on anterior face extending to anterior margin of dorsal sheets; outer margins of stalk gradually diverging from base towards basal third of furcal arms; furcal arms somewhat parallelogram-shaped, with apex (hemiductus) only slightly explanate, with apex pointing obliquely; anterior tendons inserted basad of mid length of dorsal edge of furcal arms; well-developed dorsal sheaths, narrower than widest point of lateral sheaths. ***Elytra.*** Surface even (without elevations or depressions), with sutural striae; ground punctures and systematic punctures either shallow or sharply marked (e.g., Figs 11E, 12E), similar in size and degree of impression throughout elytra, seemingly arranged in rows; serial punctures, when present (e.g., Fig. 13A, D, G), larger and deeper than ground punctures, and clearly arranged in longitudinal rows (striae); serial punctures only very slightly impressed into grooves along posterior half of elytra in striate species (e.g., *P.petilus*, *P.striatolatus*, and *P.semipubescens*; see Fig. 13); seta-bearing systematic punctures rather scarce; elytral outer margins flared, usually along entire length. Epipleura usually well developed, surface either flat or oblique, with sparse setae and irregular sculpture, anteriorly wide, gradually narrowing posteriorly, extending up to midlength of first abdominal ventrite; inner margin of epipleura only slightly indented at anterior outer corner of metepisternum; pseudepipleura usually well developed and perpendicularly positioned, ranging in width from nearly as wide as anterior portion of epipleura, to half as wide, extending up to basal half of abdomen along outer margin of elytra. Hind wings well developed. ***Legs.*** Pubescence on anterior surface of metafemora ranging from scarce and limited to anterior margin (e.g., Fig. 12C), to densely covering most surface up to apical fifth (e.g., Fig. 11G); glabrous area of metafemur with shiny and sometimes slightly reticulated surface; all femora antero-posteriorly flattened; metafemora usually with sharply marked tibial grooves. Tibiae slender, rather cylindrical; longitudinal rows of well-developed spines along pro-, meso- and metatibiae, composed of rather sharp and stout spines, slightly sparser along metatibiae; protibiae with a median longitudinal row of rather long and thick setae along anterior surface; protibial apical spurs large, extending beyond apex of protarsomere 2, sometimes reaching apex of protarsomere 3. All tarsi with five tarsomeres, bearing long apical hair-like setae on dorsal face, and spine-like or hair-like setae on ventral face of tarsomeres 2–4, sometimes also tarsomere 5; pro- and mesotarsomeres 1–4 similar in size and shape; pro- and mesotarsomere 5 approximately as long as 3–4 combined; metatarsomere 2 similar in length to metatarsomere 5; claws rather large, curved; well-developed empodium, bearing a pair of long, curved apical setae. ***Abdomen.*** Abdomen with five ventrites, rather flat to medially convex; all ventrites with uniform, dense, fine pubescence; posterior margin of fifth ventrite either rounded, truncate, or slightly emarginate, usually fringed with spine-like setae (Fig. 14C). Aedeagus (Fig. 14D–L) with basal piece as long or longer than parameres; median lobe triangular, with base nearly as wide as base of a paramere, with well-developed lateral basal apodemes; apex of median lobe variable, with a membranous to lightly sclerotized apical projection; gonopore not differentiated; parameres nearly as long as median lobe, with outer margins usually straight along basal 3/4, with setae at apex.

##### Larvae.

The immature stages are unknown.

##### Etymology.

Named from the Latin *primus*, meaning first, with the ending -*cerus*, in reference to the belonging of the genus to the Acidocerinae. To be treated as masculine.

##### Distribution.

Broadly distributed across the Guiana Shield region of South America, including Brazil (Pará), Guyana, Suriname and southern Venezuela (Amazonas, Bolívar) (Fig. 15).

##### Remarks.

The habitats occupied by members of *Primocerus* range from forested pools to seepages (Fig. 16), in an elevational range from 80 to 1950 m. Only one specimen has been collected with a flight intercept trap. Specimens of *Primocerus* are relatively rare, given that so far have only been found in low numbers of specimens per collecting event.

### Characters of taxonomic importance for *Primocerus*

The external morphology of *Primocerus* species may be considered very heterogeneous in comparison with other acidocerine genera (e.g., *Globulosis* García (see Short et al. 2017), *Quadriops* Hansen, 1999 (see Girón and Short 2017), *Crucisternum* Girón & Short, 2018).

**Body size.** Species of *Primocerus* measure approximately 3.0–3.5 mm, except for the largest species *Primocerusgrandis*, with approximately 5.0 mm.

**Elytral punctation.** Two main groups of species can be recognized by the degree of impression of the ground punctures: the smooth group (with shallowly impressed elytral punctures: *P.cuspidis*, *P.gigas*, *P.neutrum*, *P.ocellatus*; Figs 10, 11) and the punctate group (with sharply marked punctures: *P.maipure*, *P.pijiguaense*, *P.petilus*, *P.semipubescens*, *P.striatolatus*; Figs 12, 13). Within the punctate group, two groups of species can be distinguished by how evident the longitudinal rows of serial punctures are: the homogeneous group (with serial punctures only slightly distinguishable from ground and systematic punctures: *P.maipure*, *P.pijiguaense*, Fig. 12) and the striate group (with serial punctures larger than the ground punctures and clearly organized into rows: *P.petilus*, *P.semipubescens*, *P.striatolatus*; Fig. 13). In some cases (*P.petilus* (Fig. 13A, B) and *P.striatolatus* (Figs 13D, E)), the striae are very slightly impressed along the posterior half of the elytra.

**Coloration.** The general coloration of *Primocerus* specimens range from orange and reddish brown to dark brown, although there is not much variation within species groups. Teneral specimens are significantly paler than fully sclerotized ones. Specimens that have been extracted for DNA are darker. Coloration should not alone be taken as a diagnostic feature.

**Hydrofuge pubescence on metafemora.** The extent of coverage of hydrofuge pubescence of the anterior surface of the metafemora varies across species. Most species have at least the basal half of the surface covered, but in some the coverage is limited to the dorsal margin (*P.maipure*, *P.pijiguaense*, Fig. 12C, G).

**Aedeagus.** As is usual for the subfamily, the general configuration of the aedeagus (e.g., large basal piece, median lobe at base nearly as wide as base of a paramere, median lobe rather triangularly shaped, and nearly as long as parameres) is conserved across the genus, with specific diagnostic features (e.g., shape of parameres) at the species group and species level. Species groups distinguishable by characters of the elytra can also be recognized by aedeagal traits.

### Key to the species of *Primocerus*

**Table:** 

1	Elytra with ground punctures shallowly to very weakly marked (Figs 10A, E, 11A, E)	**2**
–	Elytra with ground punctures sharply marked (e.g., Figs 12A, E, 13A, D, G)	**5**
2	Body length equal to or larger than 4.0 mm	**3**
–	Body length smaller than 4.0 mm	**4**
3	Eyes in dorsal view of the head, very small (distance separating eyes 17 × the width of an eye) (Fig. 10D)	*** Primocerus ocellatus ***
–	Eyes in dorsal view of the head, of moderate size (distance separating eyes 7.5 × the width of an eye) (Fig. 10H)	*** Primocerus gigas ***
4	Posterior elevation of mesoventrite with a sharply pointed pyramidal (triangular) spine (Fig. 11C)	*** Primocerus cuspidis ***
–	Posterior elevation of mesoventrite with a curved transverse ridge, rather sharp and low (Fig. 14A)	*** Primocerus neutrum ***
5	Hydrofuge pubescence on metafemora limited to dorsal margin of anterior surface (Fig. 12C, G)	**6**
–	Hydrofuge pubescence on metafemora covering at least the entire basal third of anterior surface (Fig. 13C, F, I)	**7**
6	Apex of median lobe of aedeagus simply rounded in lateral view; base of parameres in lateral view oblique (Fig. 14F)	*** Primocerus maipure ***
–	Apex of median lobe of aedeagus carinate (dorsally projected in lateral view, Fig. 14H); base of parameres in lateral view perpendicular to longitudinal axis of aedeagus (Fig. 14H)	*** Primocerus pijiguaense ***
7	Hydrofuge pubescence covering slightly less than the basal half of the anterior surface of all femora (Fig. 13I)	*** Primocerus semipubescens ***
–	Hydrofuge pubescence covering at least basal 3/4 of the anterior surface of all femora Fig. 13C, F)	**8**
8	Elytra in dorsal view 3 × longer than wide; serial punctures not well differentiated along basal fourth of elytral striae IX and X (Fig. 13A, B)	*** Primocerus petilus ***
–	Elytra in dorsal view nearly 2.6 × longer than wide; serial punctures of elytral striae IX and X well developed along entire length (Fig. 13D, E)	*** Primocerus striatolatus ***

#### Primocerus cuspidis sp. nov.

Taxon classificationAnimaliaColeopteraHydrophilidae

http://zoobank.org/0EA5176F-B2BB-4E50-8799-C93F09B412B6

Figs 11A–D , 14L , 15B , 16A

##### Type material.

**Holotype** (♂): “VENEZUELA: Amazonas/ 5°23.207'N, 67°36.922'W; 125 m/ Tobogán de la Selva, old “Tobogancito”/ on seepage area with detritus/ 8.viii.2008; leg. A. Short, M. García, / L. Joly; AS-08-080b” (MIZA). **Paratypes (3): VENEZUELA: Amazonas**: same data das holotype (SEMC, 3).

##### Differential diagnosis.

*Primoceruscuspidis* belongs to the group of species with shallowly impressed, rather irregularly distributed, and undifferentiated elytral punctures. It can be easily distinguished among its congeners by its paler (orange) coloration, and the presence of a sharp, pyramidal (triangular) projection on the posterior elevation of the mesoventrite.

##### Description.

Body length 2.4 mm, width 1.5 mm. Body elongate oval, moderately convex (Fig. 11B). General coloration orange-brown. Elytra with ground punctures shallowly marked; serial punctures absent. Posterior elevation of mesoventrite with sharply pointed pyramidal (triangular) spine. Metafemora with hydrofuge pubescence covering basal 4/5. Apex of fifth abdominal ventrite slightly emarginate. Aedeagus (Fig. 14L) with basal piece 1.3 × longer than parameres; parameres 1.15 × longer than median lobe; distal end of parameres with anteapical constriction, apex rounded and obliquely directed; apex of median lobe widely rounded.

##### Etymology.

Named with the Latin word *cuspidis* meaning point, in reference to the sharp projection on the posterior elevation of the mesoventrite.

##### Distribution.

*Primoceruscuspidis* has only been collected at Tobogán de la Selva in the Venezuelan Amazon, at an elevation of 125 m (Fig. 15B).

##### Remarks.

The type series was collected in a flat, horizontal seepage area that was formed from water seeping from the banks of the Rio Coromoto (Fig. 16A).

#### Primocerus gigas sp. nov.

Taxon classificationAnimaliaColeopteraHydrophilidae

http://zoobank.org/D56F83E7-8B5C-4A07-87FE-30E16A936BCA

Figs 10E–H , 14I , 15A

##### Type material.

**Holotype** (♂): “VENEZUELA: Amazonas/ 0°50'N, 65°59'W; 2100 m/ Cerro de la Neblina, camp II; beetles in flight over sunlit stream/ 16:00hrs. 31.i.1985/ leg. W.E. Steiner et al.” (USNM). **Paratypes (8): VENEZUELA: Amazonas**: Same data as holotype (SEMC, USNM, 7, including DNA voucher SLE 1374); same except 0°52'N, 65°58'W, 1450 m, camp XI, 25–28.ii.1985, seine of rapids in small mountain stream, leg. P.J. & P.M. Spangler, R. Faitoute (USNM, 1).

##### Differential diagnosis.

*Primocerusgigas* is among the largest species of the genus. It can be distinguished from similarly sized species by the moderately sized eyes being separated by a distance of 7.5 × the width of an eye (Fig. 10H).

##### Description.

Body length 4.9 mm, width 2.8 mm. Body elongate oval, moderately convex (Fig. 10F). General coloration dark brown. Elytra with ground punctures shallowly marked, seta-bearing systematic punctures slightly enlarged, and serial punctures absent. Posterior elevation of mesoventrite with simple transverse ridge. Metafemora with hydrofuge pubescence covering slightly more than basal half of anterior surface. Apex of fifth abdominal ventrite truncate. Aedeagus (Fig. 14I) with basal piece nearly 1.1 × longer than parameres; parameres slightly longer than median lobe, truncate and obliquely directed at apex; apex of median lobe narrowly pointed.

##### Etymology.

Named with the Latin word *gigas* meaning giant, in reference to the large size of this species compared to most members of the genus.

##### Distribution.

*Primocerusgigas* is only known from Cerro de la Neblina in the Venezuelan Amazon, at elevations between 1450 and 2100 m (Fig. 15A).

##### Remarks.

Label data indicates the beetles were collected “in flight”, with one specimen collected by seining rapids in a mountain stream.

#### Primocerus maipure sp. nov.

Taxon classificationAnimaliaColeopteraHydrophilidae

http://zoobank.org/7C0A2BDC-E227-49C7-9940-A0F3836D50A0

Figs 12A–D , 14E, F , 15A

##### Type material.

**Holotype** (♂): “VENEZUELA: Amazonas: 5°30.623'N, 67°36.109'W; 100 m; ca. 15 Km S. of Puerto Ayacucho; rock pools on top; 14.ix.2007; leg. A. Short; AS-07-011b” (MIZA). **Paratypes (10): VENEZUELA: Amazonas**: 5°23.207'N, 67°36.922'W; 125 m/ Tobogán de la selva, old “Tobogancito”/ upstream at small slide; 12.ix.2007/ leg. M. García; AS-07-007b (SEMC, 1); “5°30.518'N, 67°36.079'W; 100 m/ ca. 15 Km S. of Puerto Ayacucho; isolated seepage/ 13.ix.2007; leg. A. Short; AS-07-009a” (SEMC, 1); same data as holotype (SEMC, 2, including DNA voucher specimen SLE 1034); same except “pools at outcrop base, AS-07-011x” (SEMC, 2); “110 m; rock outcrop pools; 8.ix.2007; leg. A. Short, M. García; AS-08-081b” (SEMC, 1); 5°48.414'N, 67°26.313'W; 80 m/ nr. Iboruwa, “Tobogancito”/ 7.viii.2008; leg. A. Short, M. García, L. Joly/ AS-08-078” (SEMC, 3).

##### Differential diagnosis.

*Primocerusmaipure* can be differentiated by the presence of sharply impressed elytral punctures, with serial punctures only slightly differentiated, longitudinally aligned (more evidently so along posterior half of elytra, Fig. 12A, B). It is very similar to *P.pijiguaense*, from which it can be distinguished by its simple median lobe and the oblique and rather angulate outer margins of the apical region of the parameres (Fig. 14E, F; apical region of median lobe dorsally keeled along apical region, and widely rounded outer margins of the apical region of the parameres in *P.pijiguaense*, Fig. 14G, H).

##### Description.

Body length 2.6 mm, width 1.5 mm. Body elongate oval, strongly convex (Fig. 12A, B). General coloration brown. Elytra with ground punctures sharply marked, with serial punctures only slightly differentiated, longitudinally aligned, more evidently so along posterior half of elytra (Fig. 12A, B). Posterior elevation of mesoventrite with simple, very lowly raised curved transverse ridge. Metafemora with hydrofuge pubescence limited to anterodorsal surface. Apex of fifth abdominal ventrite truncate. Aedeagus (Fig. 14E, F) with basal piece nearly 1.2 × longer than parameres; parameres nearly as long as median lobe, in lateral view with base oblique to longitudinal axis of aedeagus; outer margin of apical region of parameres oblique and rather angulate; apical region of median lobe simple, non-carinate.

##### Etymology.

Noun in apposition. Named after the Maipure, one of the pre-Columbian indigenous tribes that inhabited the “Spanish Guyana” region, and the language they spoke.

##### Distribution.

*Primocerusmaipure* has been collected at localities south of Puerto Ayacucho in the Venezuelan Amazon, at elevations between 80 and 125 m (Fig. 15A).

##### Remarks.

All collections of this species were made either on small seepages over granite outcrops, or in small rock pools that had formed on the outcrops.

#### Primocerus neutrum sp. nov.

Taxon classificationAnimaliaColeopteraHydrophilidae

http://zoobank.org/3129F0EC-8A73-47E3-839C-C65BDC6AAFBB

Figs 11E–H , 14D , 15A , 16C

##### Type material.

**Holotype** (♂): “VENEZUELA: Bolívar/ 6°2'10.5"N, 61°23'57.8"W; 630 m/ along La Escalera; rocky stream/ 31.vii.2008; leg. A. Short, M. García, L. Joly/ AS-08-058” (MIZA). **Paratypes (20): GUYANA: Region 8**: “4°43'49"N, 59°1'35"W; 300 m/ Iwokrama Forest, Pakatau hills/ flight intercept trap; 26–29.v.2001/ leg. R. Brooks & Z. Falin; GUY1BF01 063” (SEMC, 1); “5°0.730'N, 59°38.965'W; 585 m/ Upper Potaro camp I, c. 7 km NW Chenapau, Ridge Trail/ 11.iii.2014; leg. Short, Baca, Salisbury; GY14-0311-02A” (CBDG, SEMC, 11); “5°18.261'N, 59°50.257'W; 687 m/ Ayanganna Airstrip, trail from airstrip to Ayanganna/ forest detrital pools; 17.iii.2014/ leg. A. Short; GY14-0317-01A” (SEMC, 1); same except “18.iii.2014, GY14-0318-01B” (SEMC, 1); same except “seepage area over rocks in forest flowing into stream, GY14-0318-01C” (SEMC, 1). **SURINAME: Sipaliwini District**: “3°53.942'N, 56°10.849'W; 733 m/ CSNR: Tafelberg Summit, nr. Caiman Creek Camp/ pools in forest; 19.viii.2013/ leg. Short & Bloom; SR13-0819-05B” (SEMC, DNA voucher specimen SLE 1085). **VENEZUELA: Bolívar**: Same data as holotype (MIZA, SEMC, 8, including DNA voucher SLE 529).

##### Differential diagnosis.

*Primocerusneutrum* can be regarded as very plain in appearance, lacking remarkable features. It can be distinguished among similarly sized species with shallowly punctured elytra by its dark brown coloration and simple transverse ridge on the posterior elevation of the metaventrite.

##### Description.

Body length 2.6–3.5 mm, width 1.4–1.9 mm. Body elongate oval, moderately convex (Fig. 11F). General coloration brown. Elytra with ground punctures very shallowly marked. Posterior elevation of mesoventrite with simple curved transverse ridge. Metafemora with hydrofuge pubescence covering nearly basal 4/5 of anterior surface. Apex of fifth abdominal ventrite slightly emarginate. Aedeagus (Fig. 14D) with basal piece nearly 1.25–1.35 × longer than parameres; parameres slightly longer than median lobe, truncate to rounded and obliquely directed at apex; apex of median lobe somewhat “pinched” and narrowly pointed.

##### Etymology.

Named with the Latin word *neutrum* meaning neutral, in reference to the comparatively unremarkable appearance of the species.

##### Distribution.

*Primocerusneutrum* has been collected at the locality known as La Escalera in the Venezuelan Amazon, the Upper Potaro region and the Iwokrama Forest in Guyana, and the Tafelberg summit in Suriname. Specimens have been collected at elevations of 300–733 m (Fig. 15A).

##### Remarks.

This species has been collected in detrital pools in densely forested areas, typically associated with streams (Fig. 16C).

#### Primocerus ocellatus sp. nov.

Taxon classificationAnimaliaColeopteraHydrophilidae

http://zoobank.org/77A81130-1B8D-427F-83F8-B4F65662202C

Figs 10A–D , 15B

##### Type material.

**Holotype** (♀): “VENEZUELA: Amazonas/ Cerro de la Neblina/ Camp XII, 1950 m/ near Pico Phelps/26.ii.1985// from leaf packs and wood pieces in small stream/ leg. W. Steiner, W. Buck, B. Boom, C. Brewer” (USNM).

##### Differential diagnosis.

*Primocerusocellatus* can be easily recognized by its large size (4.4 mm), reddish coloration, and very small eyes in dorsal view (separated by a distance 17 × larger than the width of an eye).

##### Description.

Body length 4.4 mm, width 2.4 mm. Body elongate oval, strongly convex (Fig. 10B). General coloration reddish brown. Elytra with ground punctures shallowly marked, systematic punctures slightly enlarged, and serial punctures absent. Posterior elevation of mesoventrite with very lowly raised transverse ridge. Metafemora with hydrofuge pubescence covering slightly more than basal half of anterior surface. Apex of fifth abdominal ventrite rounded.

##### Etymology.

Named from the Latin word *ocellatus* which means “having little eyes”, in reference to the unusually small eyes of the species.

##### Distribution.

*Primocerusocellatus* has only been collected at Cerro de la Neblina in the Venezuelan Amazon, at an elevation of 125 m (Fig. 15B).

##### Remarks.

The single known specimen is a female that was found in “leaf packs and wood pieces in a small stream”.

#### Primocerus petilus sp. nov.

Taxon classificationAnimaliaColeopteraHydrophilidae

http://zoobank.org/46ABEE79-02F3-4D51-BCD1-6D9BB5717CAA

Figs 13A–C , 14J , 15B , 16D

##### Type material.

**Holotype** (♂): “BRAZIL: Pará: Alenquer/ 1.49292S, 54.51566W; 150 m/ Vale do Paraíso, ca. 55 km N. of Alenquer/ tiny wet rock/seepage on trail; 3.ii.2018/ leg. A. Short; BR18-0203-01G” (INPA, DNA voucher specimen SLE 1498).

##### Differential diagnosis.

*Primoceruspetilus* can be recognized by the presence of sharply impressed elytral punctures, with serial punctures well differentiated (larger and deeper than remainder punctures), longitudinally aligned to form elytral striae. It is similar to *P.semipubescens*, from which it can be differentiated by the hydrofuge pubescence of the metafemora covering basal 3/4 of the anterior surface (covering less than basal half in *P.semipubescens*). It is also very similar to *P.striatolatus*, from which it can be differentiated by the undefined elytral striae IX and X along the basal fourth of the elytra (Fig. 13B; elytral striae IX and X clearly impressed along their entire length in *P.striatolatus*, Fig. 13E).

##### Description.

Body length 3.4 mm, width 1.6 mm. Body elongate oval, moderately convex (Fig. 12A, B). General coloration dark brown. Elytra with ground punctures sharply marked, and well-defined rows of serial punctures (forming elytral striae); elytral striae very slightly impressed along posterior half of elytra. Posterior elevation of mesoventrite with simple, curved transverse ridge. Metafemora with hydrofuge pubescence covering basal 4/5 of anterior surface. Apex of fifth abdominal ventrite rounded. Aedeagus (Fig. 14J) with basal piece nearly 1.3 × longer than parameres; parameres nearly as long as median lobe (median lobe inserted further into basal piece, thus appearing shorter than parameres); apex of parameres narrowly rounded; apex of median lobe widely rounded.

##### Etymology.

Named with the Latin word *petilus* meaning slender, in reference to the relative slenderness of the body in this species.

##### Distribution.

*Primoceruspetilus* has only been collected at one locality in the north of Brazil, at an elevation of 150 m (Fig. 15B).

##### Remarks.

The single known specimen is missing the maxillary palps. It was collected on a temporary wet spot on an exposed forested rock outcrop. The rock was wet when the specimen was collected due to recent rains but was dry by the following day (Fig. 16D).

#### Primocerus pijiguaense sp. nov.

Taxon classificationAnimaliaColeopteraHydrophilidae

http://zoobank.org/BB20F156-C882-4971-8032-636A036AFD2A

Figs 12E–H , 14G, H , 15A , 16B

##### Type material.

**Holotype** (♂): “VENEZUELA: Bolívar: 6°35.617'N, 66°49.238'W; 80 m; Los Pijiguaos; morichal/rock outcrop; 14.ix.2007; leg. A. Short, M. García, L. Joly; AS-07-015” (MIZA). **Paratypes (14): VENEZUELA: Bolívar**: same data as holotype (MALUZ, SEMC, 7, including DNA voucher specimen SLE 1029); same, except “6.viii.2008, AS-08-076” (SEMC, 1); same, except “at rock outcrop, seeps and streams at night, 9.vii.2010, leg. Short, Tellez, Arias, VZ10-0709-03A” (SEMC, 1); same, except “rock pools, 7.vii.2010, VZ10-0707-01A” (SEMC, 3, including DNA voucher specimen SLE 444); “6°57.904'N, 66°36.392'W, 51 m, Outcrop ca. 15 Km NE. of los Pijiguaos, detritus flotation, 9.vii.2010, leg. Short & Tellez, VZ10-0709-01B” (SEMC, 1); “7°29'47.3"N, 65°51'44.8"W, 45 m, 2 Km E. of Río Cuchivero, rock outcrop seeps, 6.viii.2008, leg. A. Short, M. García, L. Joly, AS-08-075” (SEMC, 1).

##### Differential diagnosis.

*Primoceruspijiguaense* can be differentiated by the presence of sharply impressed elytral punctures, with serial punctures not differentiated (e.g., they look similar to the ground punctures). It is very similar to *P.maipure*, from which it can be distinguished by the dorsal keel on the apical region of the median lobe and the widely rounded outer margins of the apical region of the parameres (Fig. 14G, H; apical region of median lobe simple, non-keeled, and oblique and rather angulate outer margins of the apical region of the parameres in *P.maipure*, Fig. 14E, F).

##### Description.

Body length 2.6–3.1 mm, width 0.9–1.7 mm. Body elongate oval, strongly convex (Fig. 12F). General coloration dark brown. Elytra with ground punctures sharply marked; serial punctures not differentiated (similar to ground punctation). Posterior elevation of mesoventrite with simple, very lowly raised curved transverse ridge. Metafemora with hydrofuge pubescence limited to anterodorsal surface. Apex of fifth abdominal ventrite rounded. Aedeagus (Fig. 14G, H) with basal piece nearly 1.2 × longer than parameres; parameres nearly as long as median lobe, in lateral view with base perpendicular to longitudinal axis of aedeagus; outer margin of apical region of parameres widely rounded; apical region of median lobe with well-developed dorsal carina.

##### Etymology.

Named after Los Pijiguaos, the type locality for the species.

##### Distribution.

*Primoceruspijiguaense* has been collected at Los Pijiguaos and a few other localities north from it, at elevations between 45 and 80 m (Fig. 15A).

##### Remarks.

All collections of this species were made either on small seepages over granite outcrops, or in small rock pools that had formed on the outcrops (e.g., Fig. 16B).

#### Primocerus semipubescens sp. nov.

Taxon classificationAnimaliaColeopteraHydrophilidae

http://zoobank.org/C28EAF39-DCD6-402C-9A0D-9107CB092407

Figs 13G–I , 15B

##### Type material.

**Holotype** (♂): “GUYANA: Region VIII/ 5°17.823'N, 59°50.000'W; 684 m/ Ayanganna Airstrip, trail from Blackwater Creek Camp to Potaro River/ small forested creek with lots of detritus/ 20.iii.2014; leg. A. Short/ GY14-0320-01A” (CBDG). **Paratypes (1): GUYANA: Region VIII**: same data as holotype (SEMC, DNA voucher SLE 1079).

##### Differential diagnosis.

*Primocerussemipubescens* can be recognized by the presence of sharply impressed elytral punctures, with serial punctures well differentiated (larger and deeper than remainder punctures), longitudinally aligned to form elytral striae. It can be differentiated by the hydrofuge pubescence of the metafemora covering less than basal half of the anterior surface (covering at least basal 3/4 in *P.petilus* and *P.striatolatus*).

##### Description.

Body length 3.7 mm, width 2.0 mm. Body elongate oval, strongly convex (Fig. 13G, H). General coloration dark brown. Elytra with ground punctures sharply marked, and well-defined rows of serial punctures (forming elytral striae); elytral striae not impressed along elytra. Posterior elevation of mesoventrite with simple transverse ridge. Metafemora with hydrofuge pubescence covering less than basal half of anterior surface (Fig. 13I). Apex of fifth abdominal ventrite truncate.

##### Etymology.

Named from the Latin word *semis*, meaning half, combined with the word *pubescens*, in reference to the hydrofuge pubescence covering only half of the anterior surface of the metafemora in this species.

##### Distribution.

*Primocerussemipubescens* has only been collected around the Ayanganna airstrip in Guyana, 684–687 m in elevation (Fig. 15B).

##### Remarks.

The known specimens were collected along the margins of a sandy creek that had lots of detritus.

#### Primocerus striatolatus sp. nov.

Taxon classificationAnimaliaColeopteraHydrophilidae

http://zoobank.org/84459890-D0C8-4B48-8711-BFA9E440185D

Figs 13D–F , 14K , 15B

##### Type material.

**Holotype** (♂): “SURINAME: Sipaliwini District/ 2°58'36.7782"N, 55°24'40.986"W; 400 m/ Camp 4 (high) Kasikasima; White Rock/ seepage area on trail; 24.iii.2012/ leg. A. Short; SR12-0324-01B” (NZCS). **Paratypes (1): SURINAME: Sipaliwini District**: Same data as holotype (SEMC, 1).

##### Differential diagnosis.

*Primocerusstriatolatus* can be recognized by the presence of sharply impressed elytral punctures, with serial punctures well differentiated (larger and deeper than remainder punctures), longitudinally aligned to form elytral striae. It is similar to *P.semipubescens*, from which it can be differentiated by the hydrofuge pubescence of the metafemora covering basal 3/4 of the anterior surface (covering less than basal half in *P.semipubescens*). It is also very similar to *P.petilus*, from which it can be differentiated by the elytral striae IX and X clearly impressed along their entire length (Fig. 13E; elytral striae IX and X undefined along their basal fourth in *P.petilus*, Fig. 13B).

##### Description.

Body length 3.1 mm, width 1.6 mm. Body elongate oval, strongly convex (Fig. 13D, E). General coloration dark brown. Elytra with ground punctures sharply marked, and well-defined rows of serial punctures (forming elytral striae); elytral striae very slightly impressed along posterior half of elytra. Posterior elevation of mesoventrite with simple, curved, transverse ridge. Metafemora with hydrofuge pubescence covering basal 4/5 of anterior surface. Apex of fifth abdominal ventrite rounded. Aedeagus (Fig. 14K) with basal piece nearly as long as parameres; parameres nearly as long as median lobe (median lobe inserted further into basal piece, thus appearing shorter than parameres); apex of parameres rounded; apex of median lobe rounded.

##### Etymology.

Named from the word *stria*, combined with the Latin word *latus* meaning broad, in reference to the comparatively broad shape of the body and the clearly defined elytral striae in this species.

##### Distribution.

*Primocerusstriatolatus* has only been collected at one locality in the Kasikasima region in Suriname, at an elevation of 400 m (Fig. 15B).

##### Remarks.

Collected on a forested seepage that had lots of detritus.

### Key to the genera of New World Acidocerinae (modified from Girón and Short 2018)

**Table:** 

1	Eyes absent. Known only from a cave in Ecuador	*** Troglochares ***
–	Eyes present	**2**
2	Eyes completely divided into dorsal and ventral sections by a lateral projection of frons. Size small (<3 mm)	*** Quadriops ***
–	Eyes not divided into dorsal and ventral sections by frons. Size variable	**3**
3	Labrum concealed by clypeus, elytral margins broadly explanate. Body extremely dorsoventrally compressed	*** Helobata ***
–	Labrum not concealed by clypeus elytral margins not or at most weakly explanate. Body form variable but rarely dorsoventrally compressed	**4**
4	Elytra with distinctly impressed sutural striae. Only Neotropical region	**5**
–	Elytra without sutural striae. Both Neotropical and Nearctic	**6**
5	Posterior elevation of the mesoventrite either flat, broadly elevated or with a longitudinal elevation. Gonopore distinct and present	*** Chasmogenus ***
–	Posterior elevation of the mesoventrite with a transverse curved ridge, either sharp or reduced, or with a sharp, pyramidal (triangular) spine-like projection. Gonopore absent	*** Primocerus ***
6	Prosternum with strongly elevated median carina	*** Crucisternum ***
–	Prosternum not or only very slightly carinate or at most tectiform medially	7
7	Elytral systematic punctures very distinct, distinctly larger than surrounding ground punctation, forming five longitudinal rows along each elytron. Antennae with nine antennomeres	**8**
–	Elytral systematic punctures indistinct, usually blending with surrounding ground punctation. Antennae with eight or nine antennomeres	**9**
8	Metafemora mostly glabrous, with only few scattered setae on anterior surface	*** Ephydrolithus ***
–	Metafemora at most glabrous along apical third	*** Katasophistes ***
9	Antennae with eight antennomeres. Size small (< 3 mm)	**10**
–	Antennae with nine antennomeres. Size variable but almost always > 3 mm	**12**
10	Anterior surfaces of metafemora mostly glabrous	*** Tobochares ***
–	Anterior surfaces of metafemora densely covered by hydrofuge pubescence along basal three fourths	**11**
11	Body form circular, rounded. Size very small (1.9–2.3 mm)	*** Globulosis ***
–	Body form ovoid, parallel sided. Size exceedingly small (1.1–1.5 mm)	*** Nanosaphes ***
12	Fifth ventrite entire, without apical emargination or truncation. Maxillary palps shorter than the width of the head	*** Radicitus ***
–	Fifth ventrite with apical emargination. Maxillary palps as long or longer than the width of the head	**13**
13	Head subquadrate; eyes relatively small, separated by a distance nearly 6.5 × the maximum width of an eye; mentum and submentum roughly punctate; pubescence covering abdominal ventrites composed of long golden setae; ventral surface of tarsomeres 1–4 densely setose	*** Aulonochares ***
–	Head trapezoid; eyes moderate in size, separated by a distance nearly 4 × the maximum width of an eye; mentum obliquely strigate, submentum smooth to shallowly punctate; pubescence covering abdominal ventrites composed of short setae; ventral surface of tarsomeres 1–4 only with paired rows of denticles	*** Helochares ***

## Supplementary Material

XML Treatment for Aulonochares

XML Treatment for Aulonochares lingulatus

XML Treatment for Aulonochares novoairensis

XML Treatment for Aulonochares tubulus

XML Treatment for Ephydrolithus

XML Treatment for Ephydrolithus hamadae

XML Treatment for Ephydrolithus minor

XML Treatment for Ephydrolithus ogmos

XML Treatment for Ephydrolithus spiculatus

XML Treatment for Ephydrolithus teli

XML Treatment for Primocerus

XML Treatment for Primocerus cuspidis

XML Treatment for Primocerus gigas

XML Treatment for Primocerus maipure

XML Treatment for Primocerus neutrum

XML Treatment for Primocerus ocellatus

XML Treatment for Primocerus petilus

XML Treatment for Primocerus pijiguaense

XML Treatment for Primocerus semipubescens

XML Treatment for Primocerus striatolatus
